# Correction: Quantitative Characterization of Cellular Membrane-Receptor Heterogeneity through Statistical and Computational Modeling

**DOI:** 10.1371/journal.pone.0107095

**Published:** 2014-08-28

**Authors:** The PLOS ONE Staff

## Notice of Republication

This article was republished on August 13, 2014, to correct errors in the References section that were introduced in the typesetting process. The originally published, uncorrected article and the republished, corrected article are provided here for reference.

## Supporting Information

File S1 Originally published, uncorrected article.
(PDF)

File S2 Republished corrected article.
(PDF)

## References

[1] Weddell JC, Imoukhuede PI (2014) Quantitative Characterization of Cellular Membrane-Receptor Heterogeneity through Statistical and Computational Modeling. PLoS ONE 9(5): e97271 doi:10.1371/journal.pone.0097271 24827582 10.1371/journal.pone.0097271 PMC4020774

