# Supplementary figures and images for: Quantitative Characterization of Cellular Membrane-Receptor Heterogeneity through Statistical and Computational Modeling
Source: PLoS One. 2014 May 14;9(5):e97271. doi: 10.1371/journal.pone.0097271 (PMC4020774; doi:10.1371/journal.pone.0097271)

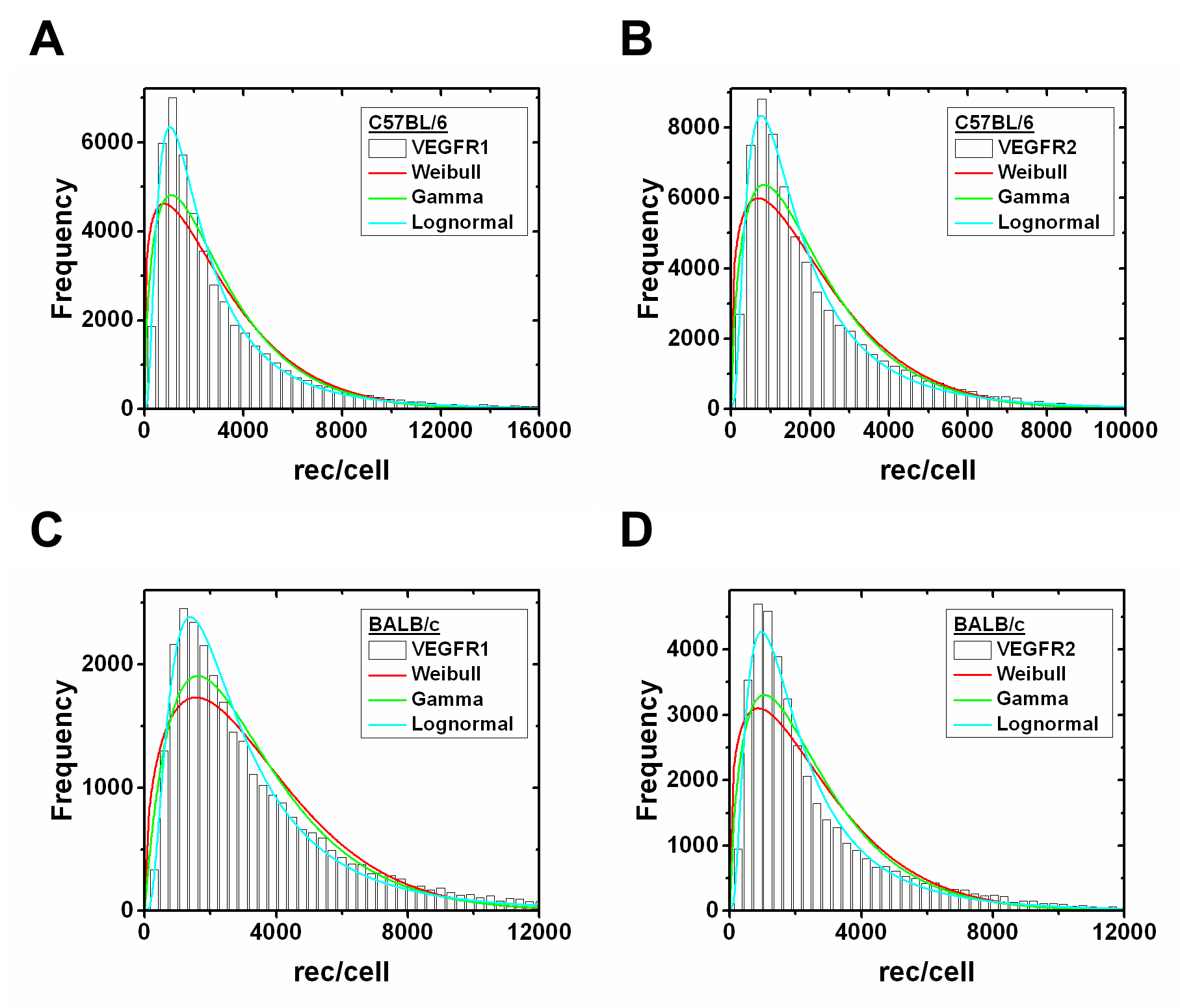

Supplement: Figure S1 — Statistical distribution fits to ex vivo receptor populations. Cell-by-cell analysis of VEGFR1/2 distributions from C57BL/6 (A-B) and BALB/c (C-D) mice. Each distribution was fit to Weibull (generalized extreme value distribution), Gamma (maximum entropy probability distribution), and lognormal (logarithm is normally distributed) probability density functions. The parameters for the best fit distributions are given in Table 2. (TIF) [file pone.0097271.s001.tif]

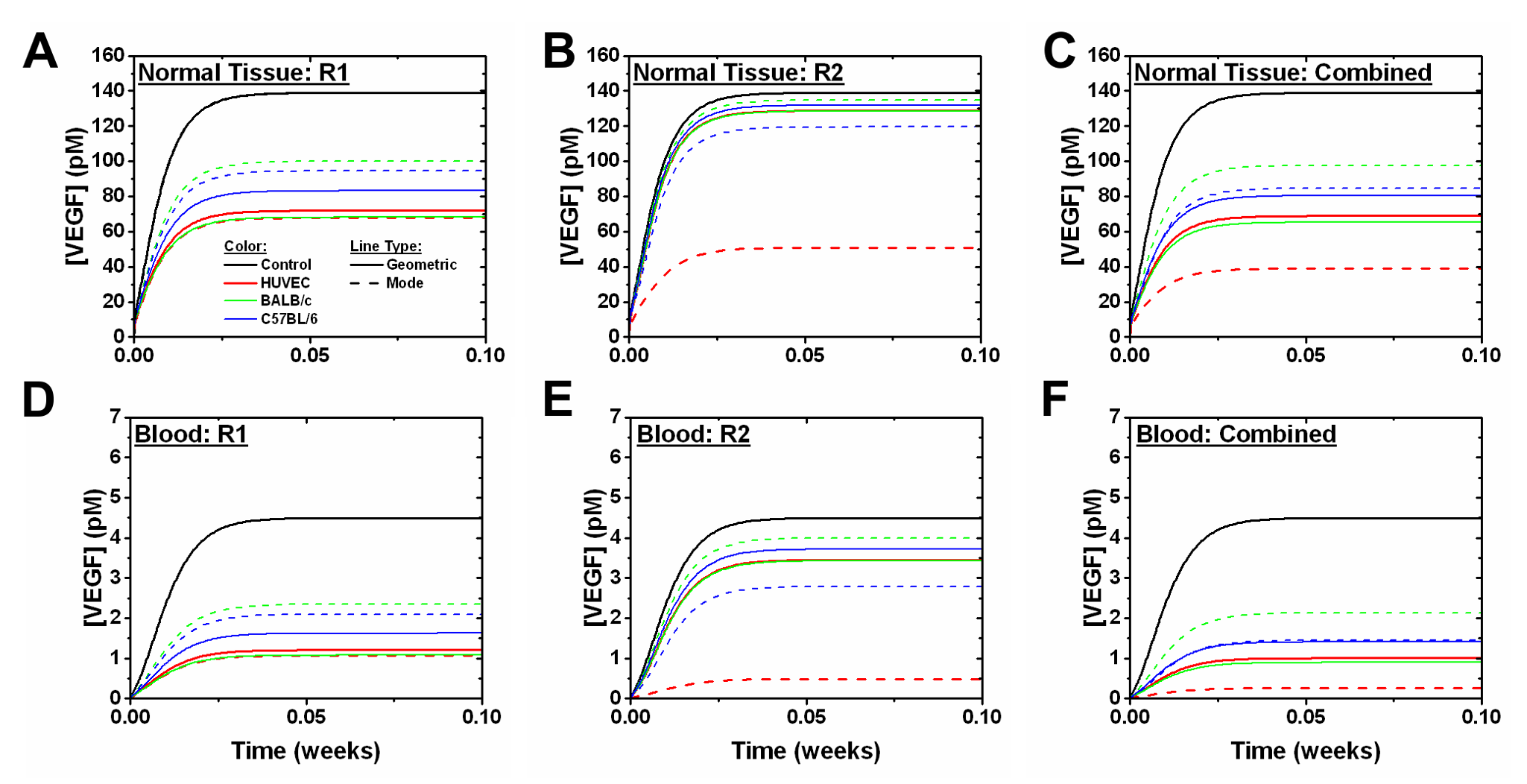

Supplement: Figure S2 — Receptor level effect on free VEGF levels in the healthy body model. Comparison of adding ex vivo or in vitro receptor levels in the healthy body model. VEGF concentration was initialized at 0 pM and simulated to steady state. The geometric mean (2,100 VEGFR1/C57, 1,540 VEGFR2/C57, 2,700 VEGFR1/BAL, 1,900 VEGFR2/BAL, 2,530 VEGFR1/HUVEC, 5,260 VEGFR2/HUVEC) and mode (1,820 VEGFR1/C57, 2,860 VEGFR2/C57, 1,700 VEGFR1/BAL, 1,200 VEGFR2/BAL, 2,720 VEGFR1/HUVEC, 11,400 VEGFR2/HUVEC) of the distributions were used. (A,D) Free VEGF response in updating VEGFR1 alone, (B,E) updating VEGFR2 alone, and (C,F) updating both simultaneously. The control reflects previously published VEGFR1 and VEGFR2 levels (1,100 VEGFR1/cell and 700 VEGFR2/cell) [23]. (TIF) [file pone.0097271.s002.tif]

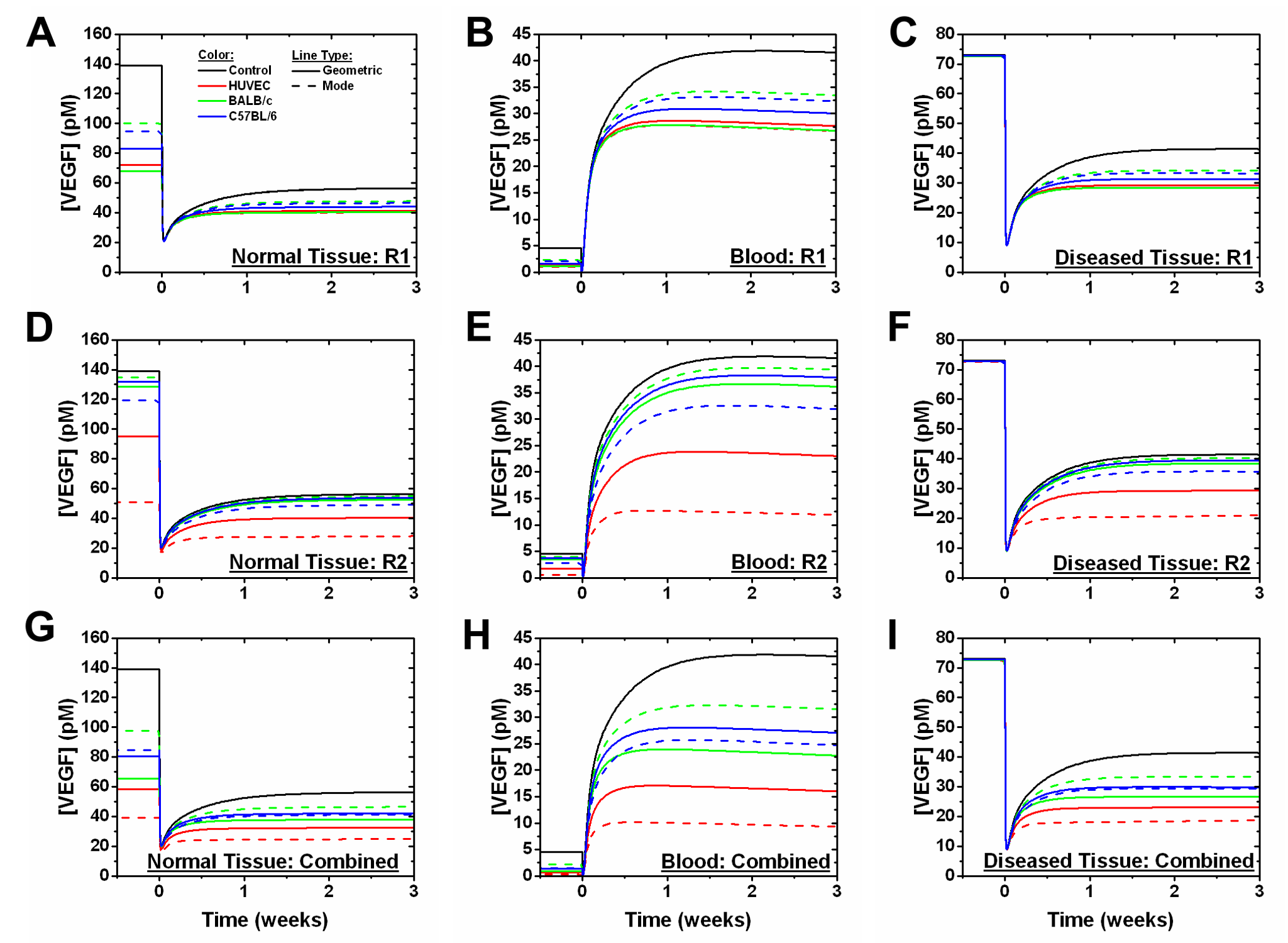

Supplement: Figure S3 — Receptor level effect on free VEGF levels in the tumor model. Comparison of adding ex vivo or in vitro receptor levels in the tumor model. An optimized anti-VEGF agent is added at t = 0 and the VEGF concentration response is simulated to 3 weeks after injection. The geometric mean (2,100 VEGFR1/C57, 1,540 VEGFR2/C57, 2,700 VEGFR1/BAL, 1,900 VEGFR2/BAL, 2,530 VEGFR1/HUVEC, 5,260 VEGFR2/HUVEC) and mode (1,820 VEGFR1/C57, 2,860 VEGFR2/C57, 1,700 VEGFR1/BAL, 1,200 VEGFR2/BAL, 2,720 VEGFR1/HUVEC, 11,400 VEGFR2/HUVEC) of the distributions were used. (A-C) Free VEGF response in updating VEGFR1 alone, (D-F) updating VEGFR2 alone, and (G-I) updating both simultaneously. The control reflects previously published VEGFR1 and VEGFR2 levels (1,100 VEGFR1/cell and 700 VEGFR2/cell) [23]. (TIF) [file pone.0097271.s003.tif]

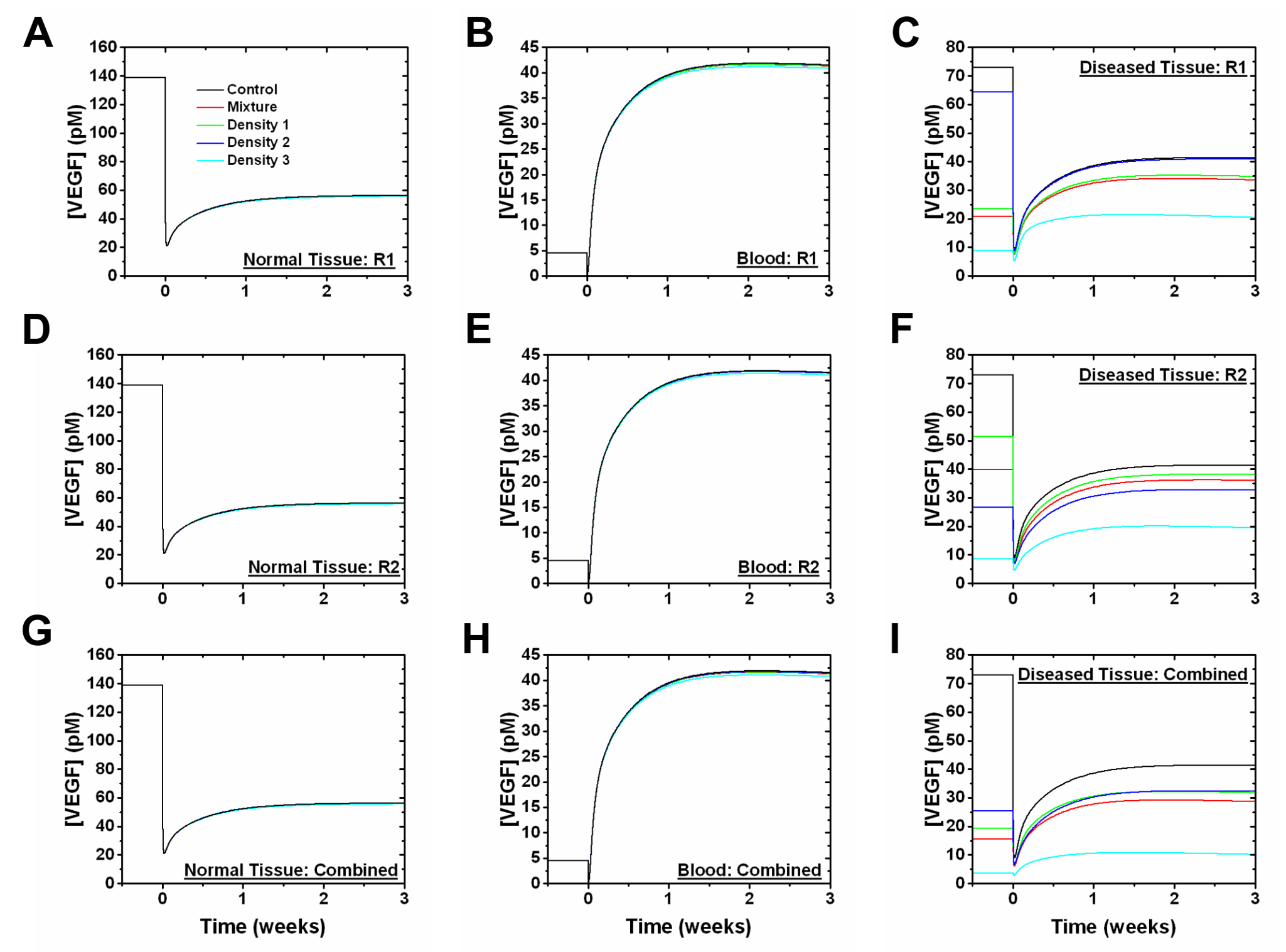

Supplement: Figure S4 — Effect of tumor cell receptor levels on anti-VEGF treatment at 3 weeks of tumor growth. Free VEGF in the normal tissue, blood, and diseased tissue compartments in response to anti-VEGF treatment after updating (A-C) VEGFR1 alone, (D-F) VEGFR2 alone, and (G-I) both receptors on the tumor cells at 3 weeks of tumor growth. “Density 1” (D1) corresponds to the Gaussian with highest weight in the mixture model, “Density 2” (D2) is the second highest weight, and “Density 3” (D3) is the lowest weight. “Mixture” was obtained by summing the geometric means of each density distribution weighted by their density in the mixture model. The geometric mean was used for all receptor distributions (3,300 VEGFR1/Mixture, 2,200 VEGFR2/Mixture, 2,900 VEGFR1/D1, 1,500 VEGFR2/D1, 1,200 VEGFR1/D2, 3,750 VEGFR2/D2, 13,250 VEGFR1/D3, 14,950 VEGFR2/D3). The control reflects previously published VEGFR1 and VEGFR2 levels (1,100 VEGFR1/cell and 700 VEGFR2/cell) [23]. (TIF) [file pone.0097271.s004.tif]

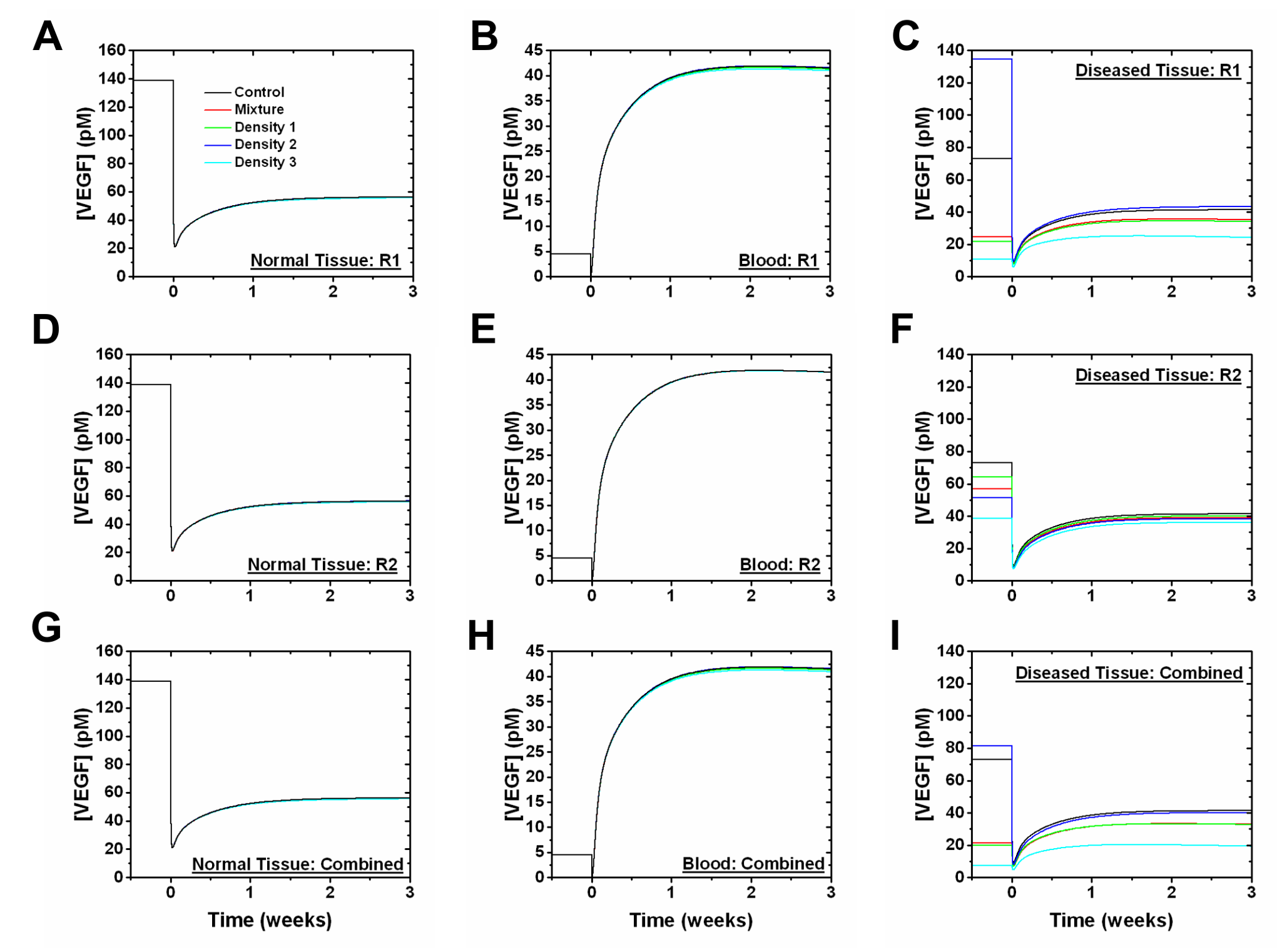

Supplement: Figure S5 — Effect of tumor cell receptor levels on anti-VEGF treatment at 6 weeks of tumor growth. Free VEGF in the normal tissue, blood, and diseased tissue compartments in response to anti-VEGF treatment after updating (A-C) VEGFR1 alone, (D-F) VEGFR2 alone, and (G-I) both receptors on the tumor cells at 6 weeks of tumor growth. “Density 1” (D1) corresponds to the Gaussian with highest weight in the mixture model, “Density 2” (D2) is the second highest weight, and “Density 3” (D3) is the lowest weight. “Mixture” was obtained by summing the geometric means of each density distribution weighted by their density in the mixture model. The geometric mean was used for all receptor distributions (2,800 VEGFR1/Mixture, 1,250 VEGFR2/Mixture, 3,150 VEGFR1/D1, 950 VEGFR2/D1, 650 VEGFR1/D2, 1,500 VEGFR2/D2, 8,500 VEGFR1/D3, 2,300 VEGFR2/D3). The control reflects previously published VEGFR1 and VEGFR2 levels (1,100 VEGFR1/cell and 700 VEGFR2/cell) [23]. (TIF) [file pone.0097271.s005.tif]
