# Supplementary material for: Quantitative Characterization of Cellular Membrane-Receptor Heterogeneity through Statistical and Computational Modeling
Source: PLoS One. 2014 May 14;9(5):e97271. doi: 10.1371/journal.pone.0097271 (PMC4020774; doi:10.1371/journal.pone.0097271)
Supplement: Table S1 — Percent of each data set defined as outliers. Percent of each complete raw data set defined as outliers, where outliers are defined using low bin search or by removing all data 3 standard deviations (STD) above the mean. The largest difference in percent defined as outliers between low bin search and 3 STD is 7.59%. (DOCX) [file pone.0097271.s006.docx]

**Table S1. Percent of each data set defined as outliers.**

|  | Low Bin Search | 3 STD Removed |
| --- | --- | --- |
| **Untreated:** VEGFR1 | 0.18 | 0.12 |
| VEGFR2 | 0.25 | 0.13 |
| VEGFR3 | 0.24 | 0.17 |
| NRP1 | 0.18 | 0.79 |
| **VEGF-A:** VEGFR1 | 0.52 | 0.1 |
| VEGFR2 | 0.08 | 0.04 |
| VEGFR3 | 8.32 | 0.73 |
| NRP1 | 0.12 | 0.67 |
| **VEGF-C:** VEGFR1 | 0.23 | 0.4 |
| VEGFR2 | 0.25 | 0.1 |
| VEGFR3 | 0.24 | 0.04 |
| NRP1 | 0.17 | 0.2 |

Percent of each complete raw data set defined as outliers, where outliers are defined using low bin search or by removing all data 3 standard deviations (STD) above the mean. The largest difference in percent defined as outliers between low bin search and 3 STD is 7.59%.
