# Supplementary material for: Correction: Quantitative Characterization of Cellular Membrane-Receptor Heterogeneity through Statistical and Computational Modeling
Source: PLoS One. 2014 Aug 28;9(8):e107095. doi: 10.1371/journal.pone.0107095 (PMC4148402; doi:10.1371/journal.pone.0107095)
Supplement: File S2 — Republished corrected article. (PDF) [file pone.0107095.s002.pdf]

Quantitative Characterization of Cellular Membrane-Receptor Heterogeneity through Statistical and Computational Modeling

Jared C. Weddell*, P. I. Imoukhuede

Department of Bioengineering, University of Illinois Urbana Champaign, Urbana, Illinois, United States of America

Abstract

Cell population heterogeneity can affect cellular response and is a major factor in drug resistance. However, there are few techniques available to represent and explore how heterogeneity is linked to population response. Recent high-throughput genomic, proteomic, and cellomic approaches offer opportunities for profiling heterogeneity on several scales. We have recently examined heterogeneity in vascular endothelial growth factor receptor (VEGFR) membrane localization in endothelial cells. We and others processed the heterogeneous data through ensemble averaging and integrated the data into computational models of anti-angiogenic drug effects in breast cancer. Here we show that additional modeling insight can be gained when cellular heterogeneity is considered. We present comprehensive statistical and computational methods for analyzing cellomic data sets and integrating them into deterministic models. We present a novel method for optimizing the fit of statistical distributions to heterogeneous data sets to preserve important data and exclude outliers. We compare methods of representing heterogeneous data and show methodology can affect model predictions up to 3.9-fold. We find that VEGF levels, a target for tuning angiogenesis, are more sensitive to VEGFR1 cell surface levels than VEGFR2; updating VEGFR1 levels in the tumor model gave a 64% change in free VEGF levels in the blood compartment, whereas updating VEGFR2 levels gave a 17% change. Furthermore, we find that subpopulations of tumor cells and tumor endothelial cells (tEC) expressing high levels of VEGFR (>35,000 VEGFR/cell) negate anti-VEGF treatments. We show that lowering the VEGFR membrane insertion rate for these subpopulations recovers the anti-angiogenic effect of anti-VEGF treatment, revealing new treatment targets for specific tumor cell subpopulations. This novel method of characterizing heterogeneous distributions shows for the first time how different representations of the same data set lead to different predictions of drug efficacy.

Citation: Weddell JC, Imoukhuede PI (2014) Quantitative Characterization of Cellular Membrane-Receptor Heterogeneity through Statistical and Computational Modeling. PLoS ONE 9(5): e97271. doi:10.1371/journal.pone.0097271

Editor: Daotai Nie, Southern Illinois University School of Medicine, United States of America

Received January 8, 2014; **Accepted** April 16, 2014; **Published** May 14, 2014

Copyright: © 2014 Weddell; Imoukhuede. This is an open-access article distributed under the terms of the Creative Commons Attribution License, which permits unrestricted use, distribution, and reproduction in any medium, provided the original author and source are credited.

Funding: This work was supported by:NIH National Cancer Institute Research Supplements to Promote Diversity; American Cancer Society, Illinois Division Basic Research Grant. The funders had no role in study design, data collection and analysis, decision to publish, or preparation of the manuscript.

Competing Interests: The authors have declared that no competing interests exist.

* Email: Jwedde3@illinois.edu

Introduction

Drug resistance is one of the largest challenges in providing effective cancer treatment, and cellular heterogeneity is a main component of drug resistance [1]. Cellular heterogeneity can regulate systemic response, and mapping these heterogeneities is a grand challenge in biomedical research [2]. Heterogeneity also represents a challenge in the emerging field of personalized medicine since variability in patient populations can result in differential therapeutic outcomes [3]. Recent analyses of breast cancer cell lines and xenografts on the genetic and proteomic levels have offered significant insight into tumor heterogeneity [4,5]. Similarly, genetic screens of patient tumor samples have highlighted the challenge of tumor heterogeneity in both personalized medicine and biomarker development [6]. Understanding how to characterize cellular heterogeneity will help surmount drug resistance challenges and develop more effective cancer treatment approaches.

Recent efforts to characterize heterogeneity have applied optical biosensors [7–9]. We have recently optimized conditions for phycoerythrin (PE)-antibody based labeling and profiling the vascular endothelial growth factor receptors (VEGFR) on endothelial cells, *in vitro* [10] and *ex vivo* [11–13], with fluorescence calibration to commercially available PE beads [14,15]. These data showed significant cell-by-cell variation in surface receptor levels across similar cell populations. We have also developed new quantum dot beads for quantitative calibration of heterogeneity [16]. The development of these tools to characterize heterogeneity has resulted in a need for better data analysis and model incorporation methods.

Defining standards for statistically characterizing heterogeneous data often requires presuppositions on the type of statistical distribution. In analyzing flow cytometry data, Boedigheimer and Ferbas automated data gating (selection). Using expectation maximization, they modeled the data as a mixture of Gaussian distributions [17]. Various commercial software automatically gate with a predefined bivariate normal or *t*-distributions [18]. However, analyzing heterogeneous data with such presuppositions could neglect essential features. As such, methods for statistically characterizing heterogeneous data with no predisposition on the data distribution are needed.

Systems biology offers useful approaches for gaining both data-driven and *in silico* insight into heterogeneous biological systems. In particular, *in silico* models have applied sensitivity analysis to probe population outliers as a proxy for heterogeneity: Schoeberl *et al* showed that increasing the number of epidermal growth factor receptors (EGFR) ten-fold in the presence of external epidermal growth factor (EGF) resulted in approximately a 3-fold decrease of the amount of extracellular signal-regulated kinase (ERK) phosphorylated downstream the signal transduction pathway within one hour [19]. Saterbak *et al* showed that heterogeneity in membrane receptors-cell adherence significantly alters cell detachment kinetics to an applied shear force [20]. While these and other computational models have shown the importance to account for receptor heterogeneity, well defined methods have not been developed for integrating experimental heterogeneity into deterministic models.

In this study, we present comprehensive statistical and computational methods for analyzing cellomic data sets and integrating them into deterministic models. Here we show that fitting protein data to statistical distributions provides useful parameters for describing cellular populations. We show how these various cellular population representations affect the vascular endothelial growth factor (VEGF) distribution throughout a whole-body model, anti-VEGF treatment efficacy, and how these representations may affect anti-VEGF treatment at different time points during tumor growth. Additionally, we mathematically describe the necessity to target treatment techniques to tumor cell subpopulations.

Materials and Methods

Experimental receptor data

VEGFR1, -R2, -R3, and neuropilin-1 (NRP1) levels were quantified on human umbilical vein endothelial cells (HUVEC) acquired from individual donors (Lonza, Walkersville, MD and Stem Cell Technologies, Vancouver, Canada), as previously described [10]. VEGFR1 and -R2 levels were quantified on primary mouse endothelial cells, which were freshly isolated from gastrocnemius and tibialis anterior of male and female 8–14 week old C57BL/6 (Charles River and NCI) and BALB/c (NCI) mice, as previously described [11]. VEGFR1 and -R2 were quantified on tumor endothelial cells and tumor cells obtained from MDA-MB-231 xenograft studies, as previously described [13]. These MDA-MB-231 cells were kindly provided by Dr. Zaver M. Bhujwalla (Johns Hopkins University) with the following details about the cell line: MDA-MB-231 breast cancer cells are purchased from the American Type Culture Collection (ATCC) and used within 6 months of obtaining them from ATCC; the cell line is tested and authenticated by ATCC by two independent methods; the ATCC cytochrome C oxidase I PCR assay and short tandem repeat profiling using multiplex PCR, this is as previously described [13]. No experimental data was acquired in this study. Ethics committee approval for data used here can be found in our previous publications [10,11,13].

Computational Models

The Popel laboratory has developed a whole-body VEGF kinetic and transport model necessary for building VEGF-mediated angiogenesis models [21,22]. We and others recently advanced this model to include experimentally quantified VEGFR levels on tissue, to explore the pharmacokinetics and pharmacodynamics associated with an anti-VEGF drug (Fig. 1), and how this drug influences systemic, tissue, and tumor VEGF levels [23]. This model contains three compartments, normal tissue, blood, and diseased tissue, to model whole-body VEGF kinetic interactions and transport between compartments, developed from experimental observations. The model predicts VEGF distribution throughout the body in response to the anti-VEGF recombinant humanized monoclonal antibody bevacizumab. This model previously showed that the anti-VEGF treatment outcome is dependent on the tumor microenvironment, such as receptor expression, and may be affected by heterogeneities across patients. Receptor insertion and trafficking is simplified by assuming a constant receptor levels on the cell surface. All receptors are assumed as pre-dimerized homodimers, and this model does not account for dimerization kinetics or heterodimers. Model components, assumptions, and experimental bases are described in detail in previous publications [21–23]. A healthy model excluding the tumor compartment was also developed, which includes all of the components in the schematic above the "Healthy Body cutoff" line (Fig. 1). Luminal and abluminal surfaces of endothelial cells are assumed to have the same receptor levels. Cellular heterogeneity is explored within normal physiology and through anti-VEGF drug treatment response using these models. An anti-VEGF agent is administered in the model as an injection into the blood at time $t=0$, and all simulations continue to 3 weeks after anti-VEGF injection [23]. VEGF levels in response to altering model parameters are measured as they directly correlate with angiogenesis occurrence.

Applying receptor values to the computational model

Four methods are implemented to condense the data distribution into a single VEGFR surface level: median, mode, geometric mean, and arithmetic mean. These four methods were chosen as they all have different biases towards choosing a representative value from the population. The mode and median are biased towards values that are repeated frequently, the arithmetic mean is biased towards the tail of a population, whereas the geometric mean creates a balance between the frequency and the range of values. VEGFR levels are updated from the extracted value, and free VEGF in each compartment is simulated up to 3 weeks after injecting an anti-VEGF drug. Updates are done for VEGFR1 alone, VEGFR2 alone, or both using the same extraction method. Free VEGF using parameter updates is compared to control which reflects VEGFR levels previously published (1,100 VEGFR1/cell and 700 VEGFR2/cell) [23].

Results

Developing the low bin search method

Optimal bin size for plotting a data set as a histogram is determined by fitting to three statistical distributions: Weibull, Gamma, and lognormal, using a range of bin sizes. These were chosen due to their characteristic properties: Weibull is a special case of the generalized extreme value distribution which approximates the maxima of a finite sequence of random variables; Gamma is the maximum entropy probability distribution which chooses the unknown distribution that exhibits the highest entropy; and lognormal fits a distribution whose logarithm follows a Gaussian. The three statistical distributions are all two parameter distributions, and for a given value x their probability density functions $f(x)$ are as follows:

Weibull:

$$f(x;\lambda,k)=\begin{cases}\frac{k}{\lambda}\left(\frac{x}{\lambda}\right)^{k-1}e^{-(x/\lambda)^k} & x\geq 0\\ 0 & x<0\end{cases} \quad (1)$$

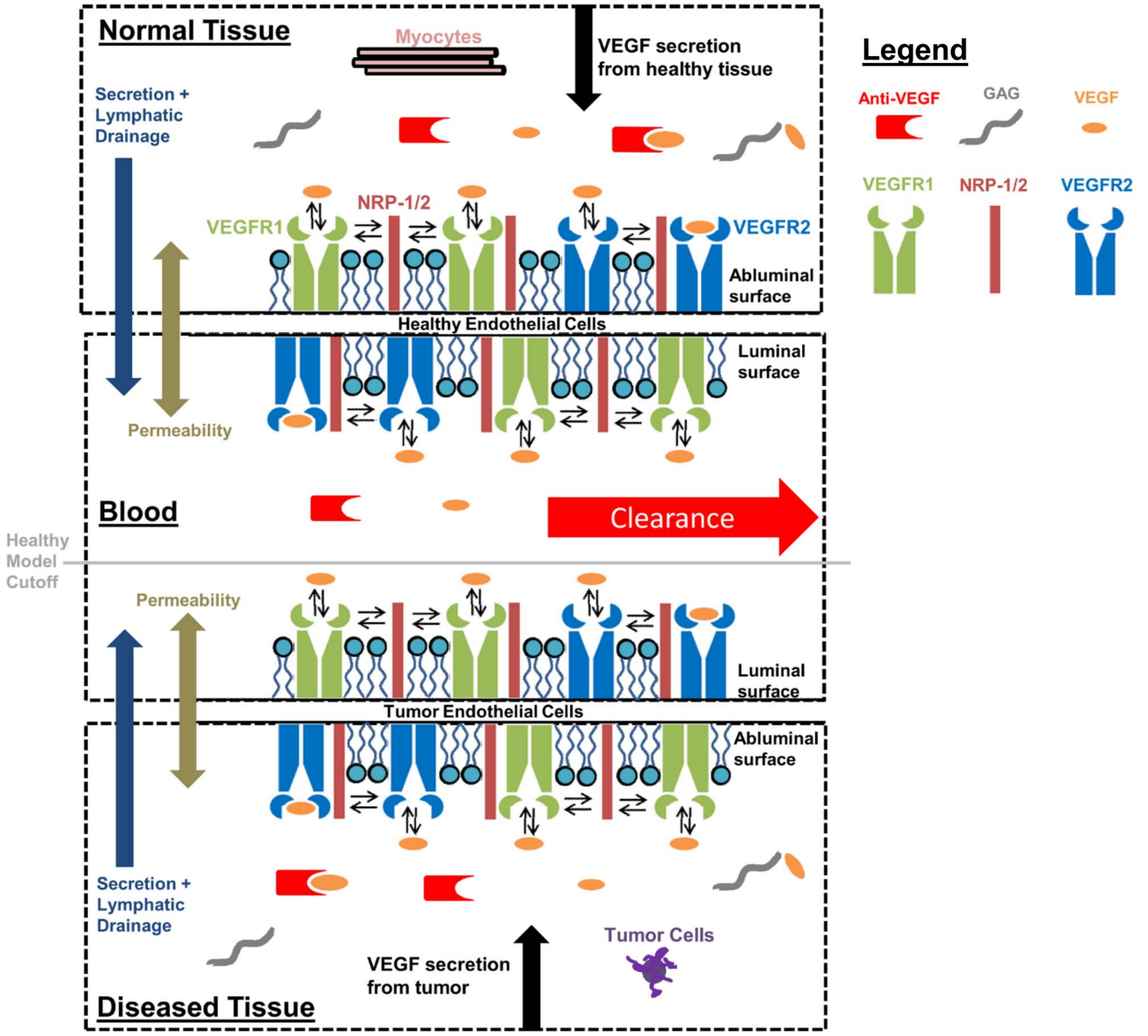

Figure 1. Schematic of the compartments and major components included in the VEGF model. The VEGF model contains a normal tissue, blood, and diseased tissue compartment. VEGF interacts anti-VEGF, GAG chains, and with its receptors VEGFR1/2, on tumor cells and Myocytes, as well as NRP1/2 on endothelial cells. The luminal surface of the endothelial cells resides in the blood compartment, whereas the abluminal side resides in the normal tissue compartment on healthy endothelial cells and the diseased tissue compartment on tECs. VEGF is secreted by the tumor in the diseased compartment and tissue in the normal tissue compartment. VEGF is also secreted and lymphatically drained to the blood compartment where it is cleared. VEGF is also permeable through the blood vessel wall. A thorough description of the model containing a complete list of species and parameters can be found in Finley *et al* [23]. The tumor model contains all of the shown components, while the healthy model contains only those above the ''healthy model cutoff'' line.
doi:10.1371/journal.pone.0097271.g001

Gamma:

$$f(x;\lambda,k)=\begin{cases}\frac{x^{k-1}e^{-\frac{x}{\lambda}}}{\lambda^k\Gamma(k)} & x\geq 0\\ 0 & x<0\end{cases} \quad (2)$$

Lognormal:

$$f(x;\mu,\sigma)=\begin{cases}\frac{1}{x\sigma\sqrt{2\pi}}e^{\frac{-(\ln x-\mu)^2}{2\sigma^2}} & x>0\\ 0 & x\leq 0\end{cases} \quad (3)$$

where k is the shape parameter, λ is the scale parameter, $\Gamma(k)$is the gamma function evaluated at k,$\mu$is the mean, and$\sigma$is the standard deviation. The two parameters of each distribution that best describe the experimental data are determined using MATLAB. Next it is determined which of the three statistical

distributions best represents the data by minimizing the sum of squared errors (SSE). The SSE for all three distributions is calculated by

$$SSE = \sum_{i=1}^{n} (y_i - \overline{y_i})^2 \quad (4)$$

where n is the total number of bins in the histogram, $\overline{y_i}$ is the number of elements in the ith bin, and y_i is the statistical distribution evaluated at x_i, the center of the ith bin. For example, for a bin that ranges between 10 and 20 surface receptors,$y_i$is the value given by the statistical distribution evaluated at 15 surface receptors. The histogram is the experimentally obtained receptor data, and thus $\overline{y}$ is the true value while y_i is the approximated value from fitting the statistical distribution. The SSE was used to make two decisions: (1) Decide which statistical distribution fit the data best. The best fitting distribution had the lowest SSE amongst the three distributions given the same number of bins. However, the best fitting distribution is not dependent on the number of bins. Next, (2) decide the optimal number of bins to define the histogram. The optimal bin number was determined by minimizing the SSE the best fit distribution gave across a range of histograms with different numbers of bins. Thus, if the Gamma distribution gave the best fit, the optimal bin number$B$would be determined as

$$B = \min\left(SSE_i^{Gamma}\right) \forall i \in \{5,6,...,m\} \quad (5)$$

where $SSE_i^{Gamma}$is the SSE given by the Gamma distribution fit using a number of bins i, where $i$ranges from 1 to m. Note that m is defined as the maximum number of bins desired to test, and we used $m = 500$ bins. Also note that i starts at 5 bins as starting with a smaller number of bins is not practical for representing the data.

The histogram is using the optimal bin number is then made. Each bin of index j in the histogram is centered at a_j, has a width w, and contains a number of cells b_j. Physically, a_j is the median surface receptor level in bin j and b_j is the number of cells in the population whose surface receptor level falls in the range of the bin $\left[a_j - \frac{w}{2}, a_j + \frac{w}{2}\right)$. Note that all bins have the same width $w$and that a_j and w are defined automatically once the optimal bin number is specified. The number of cells b_j in a bin is determined by

$$b_j = \sum_{i=1}^{C} cell_i^r \forall r \in \left\{ \left(a_j - \frac{w}{2}\right) \le r \le \left(a_j + \frac{w}{2}\right) \right\} \quad (6)$$

where $C$is the total number of cells in the data set and $r$is surface receptor level the index i cell contains. We define that if $r$is within the bin range $cell_i^r = 1$, otherwise $cell_i^r = 0$. For example, a bin with $a_j = 7, w = 4$, and $b_j = 3$ means 3 cells in the population had between 5 and 9 surface receptors.

After defining the optimal bin number, we define outliers to account for unwanted data obtained from flow cytometry, such as surface receptor levels outside the physiological range. As the data is heterogeneous, we define a cutoff method for specifying outliers that utilizes the data shape and the optimal bin number. The cutoff is determined by the bin with lowest a_j that also meets the following two criteria:

(1) The number of cells in the bin is less than 1% the number of cells in the largest bin. For example, if the largest bin has 500 cells, the cutoff bin must have less than 5 cells.

(2) The neighboring bins have a number of cells less than 1% the number of cells in the largest bin.

The first criterion ensures the cutoff bin has low occurrence probability, while the second criterion ensures uniqueness. Mathematically, the cutoff bin is chosen when the following conditions are met:

$$\text{Cutoff} = \left\{ \min\left(a_j\right) | b_{j-1} < 0.01 \cdot b_m,\ b_j < 0.01 \cdot b_m,\ b_{j+1} < 0.01 \cdot b_m \right\} \quad (7)$$

where b_j is the number of cells contained in the bin of index j, b_{j-1} and b_{j+1} are the number of cells contained in the neighboring bins, and b_m is the number of cells in the largest bin. Once the cutoff bin is determined, data within that bin and all bins to the right are removed. The cutoff point is defined using the histogram with the optimal bin number. The optimal binning minimizes the SSE that the best fit distribution gives across a range of histograms with different numbers of bins. Thus, optimal binning ensures outliers are best defined using the best data representation. Henceforth, we refer to this method as the "low bin search" method. Note that the geometric mean, arithmetic mean, mode, and median are taken from the complete data set, minus outliers, not from the binned data. For this study, we were only interested in defining right hand side outliers, and only bins to the right of the largest bin are cutoff candidates. This is due to there being no negative data, in addition to cells that either have no or low expression levels. Previous studies have shown that low VEGFR expression does occur in tip-stalk cell selection, where activation of Notch1 receptors downregulates VEGFRs [24,25]. Thus, we did not consider left hand outliers, as our data is consistent with these experimental findings. In summary, low bin search (1) determines the best fitting distribution; (2) finds the optimal bin number using the best fit distribution; (3) defines a cutoff point using the optimal bin number.

Robustness of low bin search

Two bootstrapping methods were performed using the VEGFR1 data obtained from C57BL/6 mice to observe the robustness of low bin search. First, low bin search was performed on a random data sample to determine the cutoff value (Fig. 2A) and the geometric mean (Fig. 2B) after defining outliers. Second, randomly selected samples within the complete data set were increased by 20% and low bin search determined the cutoff value (Fig. 2C) and geometric mean (Fig. 2D). Sample sizes used were 1,000, 5,000, and 10,000, and 100 trials were run for each sample size and bootstrapping pair. The cutoff values found from the random sampling fall within a range of 10,000 receptors/cell, whereas the geometric means fall within a range of 130 receptors/cell. The cutoff values found after increasing values by 20% fall within a range of 2,500 receptors/cell, whereas the geometric means fall within a range of 20 receptors/cell. The smallest and largest geometric means for each sampling size and bootstrapping pair were used as parameters in the model to determine if bootstrapping caused differences in predicted free VEGF levels (Table 1). The largest difference in free VEGF in the diseased tissue compartment, compared to the complete data set, from all these tests deviated by 5.24%. This deviation is negligible, and low bin search is therefore considered robust for our purposes.

Defining outliers in the HUVEC data

We developed and tested a methodology for defining outlying data by examining cell-by-cell VEGFR3 data treated with 1 nM VEGF-A_{165} (Fig. 3). These data naturally exhibit a heavy tail, which prevents fitting to Weibull and Gamma distributions (Fig. 3A). After removing outliers using low bin search, described in the Materials and Methods and a cutoff of 2%, which gave the cutoff at 22,000 receptors/cell, the tail was reduced and the Weibull and Gamma distributions better fit the data (Fig. 3B). A common data cut-off approach of defining outliers as three standard deviations above the mean [26] also resulted in a worse fit than that given by low bin search to Weibull and Gamma distributions (Fig. 3C). We found that the minimum SSE for the Gamma distribution with no outliers removed was approximately 95 times larger than the minimum SSE after implementing low bin search. Removing three standard deviations above the mean gave an approximately 5 times larger minimum SSE than that using low bin search. Likewise for the Weibull distribution, removing no outliers gave an approximately 64 times larger, and removing 3 standard deviations above the mean gave an approximately 4 times larger minimum SSE than that using low bin search. Comparing the number of data points defined as outliers between low bin search and three standard deviations shows that low bin search removed 7.59% more raw data, at most, over all the data sets (Table S1).

HUVEC surface receptor data

The VEGFR1, -2, -3, and NRP1 surface expression on HUVECs represent distributions that are not normally distributed (Fig. 4). As such, this data is not represented best by the arithmetic mean in computational modeling. Therefore, we examined fittings to Gamma, Weibull, and lognormal distributions (Fig. 4). Important features revealed by these fittings include the following: VEGFR1, -2, and -3 distributions are positively skewed and best fit to the lognormal distribution (Fig. 4A-C); whereas NRP1 is best fit to the Gamma distribution (Fig. 4D). For comparison, we fit the receptor data to the Gaussian distribution (Fig. 4). For VEGFR1, the minimum SSE given by the Gaussian fit was approximately 1,700 times larger than the minimum SSE given by the lognormal fit. Likewise for VEGFR2, the Gaussian SSE was approximately 440 times larger than the lognormal SSE, approximately 130 times larger for VEGFR3, and approximately 45 times larger than the Gamma distribution fit to NRP1. NRP1 levels are an order of magnitude higher than any VEGFR level (Table 2). VEGFR1/2 surface expression on C57BL/6 and BALB/c mice were also fit to Gamma, Weibull, and lognormal distributions (Fig. S1, Table 2). For several distributions, such as VEGFR2 on HUVECs, the mode is larger than the other three representative parameters. This is because these parameters were taken from the complete data set and not the binned data; thus, the mode may not necessarily be contained in the largest bin. Other than these cases, the arithmetic mean is the largest parameter as it has the most bias towards the heavy tail of distributions. Since the geometric mean accounts for the tail without biasing it, we choose it as the best representing parameter. For comparing these parameters, we will compare the geometric mean to the mode as those have the largest discrepancy in value.

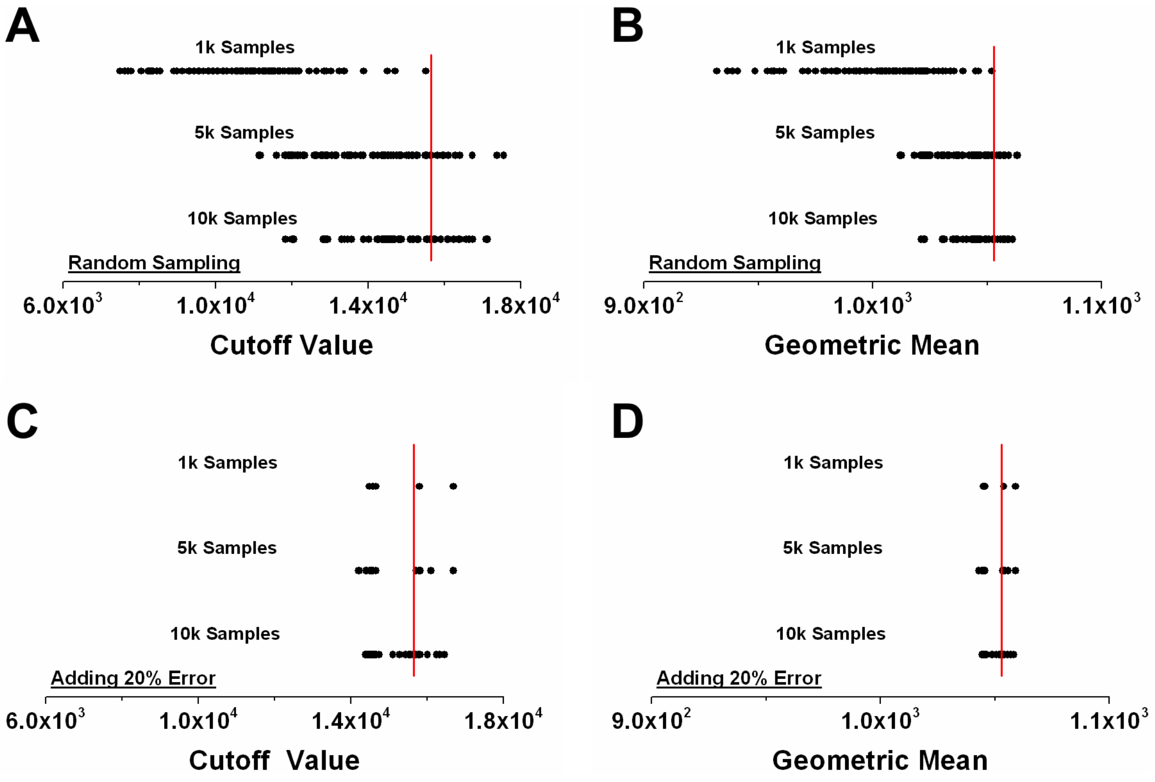

Figure 2. Bootstrapping low bin search. The cutoff value and geometric mean determined from low bin search (A-B) applied to a random subset (A-B) and after adding 20% error to random data (C-D) using the VEGFR1 data set from C57BL/6 mice. Sample sizes are 1,000, 5,000, and 10,000. Each sample size and bootstrapping method pair contains data from 100 trials, however, due to point clustering, the plots may appear to contain less trials. Red lines show the cutoff (15,642 rec/cell) and geometric mean (1,053 rec/cell) given by low bin search using the complete data set.
doi:10.1371/journal.pone.0097271.g002

Table 1. Bootstrapping the low bin search method.

Sample Size	Complete Data Set	1,000 Max	1,000 Min	5,000 Max	5,000 Min	10,000 Max	10,000 Min
Sampling	31.28	31.29	32.92	31.15	31.80	31.18	31.68
Add 20%	31.28	31.20	31.38	31.20	31.40	31.21	31.38

Bootstrapping by either (1) running low bin search on random samples of the data set or (2) increasing randomly selected samples in value by 20% using the VEGFR1 data from C57BL/6 mice. Values in the table indicate free VEGF (pM) in the diseased compartment 3 weeks after anti-VEGF treatment using the maximum and minimum geometric means obtained from 100 trials of each sample size shown in Figure 2. Free VEGF using the geometric mean obtained from the complete data set is shown to compare how much bootstrapping perturbs the system output.
doi:10.1371/journal.pone.0097271.t001

VEGFR1 affects both initial and quasi-steady state free VEGF concentrations

VEGFR1 and VEGFR2 levels on healthy endothelial cells were updated within the tumor model to the levels found on endothelial cells of C57BL/6 mice. Changes were made sequentially and in combination to observe the sensitivity of the drug treatment to each specific receptor (Fig. 5). VEGFR1 is a greater effector of free VEGF levels in each compartment: we observe a 40% decrease in initial free VEGF levels, before anti-VEGF treatment, in the normal tissue compartment. This occurs when VEGFR1 levels on healthy endothelial cells are updated from 1,100 VEGFR1/cell (550 abluminal VEGFR1/cell and 550 luminal VEGFR1/cell), which was used in the previous model [23] and represented ensemble averaged VEGFR1 levels on both C57BL/6 and BALB/c endothelial cells [11], to 2,110 VEGFR1/cell (1,055 abluminal VEGFR1/cell and 1,055 luminal VEGFR1/cell), which represents the C57BL/6 geometric mean (Table 2). VEGFR1-mediated decrease in initial free VEGF concentrations in the normal tissue compartment gave a 40% decrease relative to control, compared to only a 5% decrease when updating VEGFR2 alone. When both VEGFR1 and VEGFR2 are updated on the healthy endothelial cells, we observe a 42% decrease in the initial free VEGF levels, further confirming that VEGFR1 has a greater effect on free VEGF levels (Fig. 5A). We also observe a 1.7-fold decrease in initial free VEGF levels relative to control. These updates to the normal tissue compartment also affected the blood compartment, where free VEGF levels were decreased by 64% when updating VEGFR1 alone, decreased by 17% when updating VEGFR2 alone, and decreased by 68% when both receptors are updated (Fig. 5B). Updates to either or both VEGFR1 and VEGFR2 had no noticeable effect on initial free VEGF in the diseased tissue compartment (Fig. 5C). Free VEGF reached quasi-steady state 3 weeks after anti-VEGF administration. In the normal tissue compartment, this quasi-steady state level is decreased from control levels by 22% when updating VEGFR1 alone. The quasi-steady state free VEGF levels only exhibited a modest decrease of 4% when updating VEGFR2 alone, and a total decrease of 25% when both receptors are updated (Fig. 5A). In the blood compartment, this quasi-steady state level is decreased from control levels by 28% when updating VEGFR1 alone, decreased only 9% when updating VEGFR2 alone, and had a total decrease of 35% when both receptors are updated (Fig. 5B). In the diseased tissue compartment, this quasi-steady state level is decreased from control levels by 25% when updating VEGFR1 alone, decreased only 5% when updating VEGFR2 alone, and had a total decrease of 28% when both receptors are updated (Fig. 5C).

Representation of VEGFR levels effects model predictions

Since slight changes in VEGFR levels cause varying effects on the initial and quasi-steady state free VEGF concentrations, we next survey the data representation approaches with regards to their predicted anti-VEGF drug treatment efficacy. This was achieved by examining how updating both VEGFR1 and VEGFR2 controls the fold change in free VEGF levels after anti-VEGF injection, which we define as: $\frac{[VEGF]_{t=\text{quasi-steady state}}}{[VEGF]_{t=0}}$. An increase in free VEGF levels after anti-VEGF injection is defined as a positive change, while a decrease is defined as a negative change. We observe a larger magnitude change in free VEGF in the blood and normal tissue compartments relative to control when the model is updated to reflect the VEGFR1 and VEGFR2 mode, median, geometric mean, and arithmetic mean on healthy endothelial cells from C57BL/6 (Fig. 6A) and BALB/c

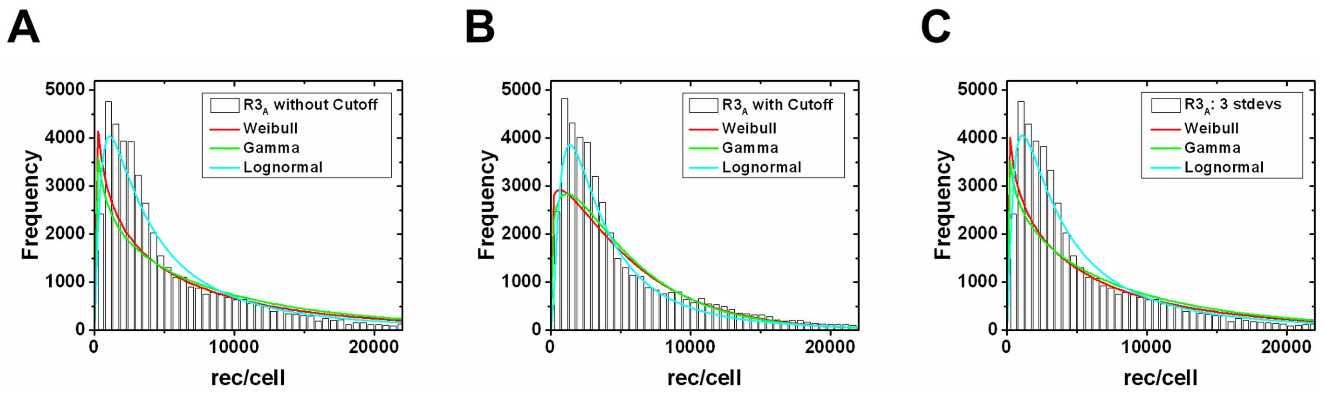

Figure 3. Effectiveness of low bin search. Effect of low bin search on distribution fitting using HUVEC data of VEGFR3 treated with VEGF-A. (A) Weibull and Gamma distributions were unable to fit the raw data. (B) After implementation of the cutoff method, Weibull and Gamma distributions were able to fit the data. (C) Comparison to removing all data 3 standard deviations above the mean, which is also unable to properly fit the data. Goodness of fit was measured by the combined sum of squared error of each statistical distribution.
doi:10.1371/journal.pone.0097271.g003

(Fig. 6B) mice. All receptor levels give a negative free VEGF fold change in the diseased tissue compartment, which indicate decreased VEGF levels.

We observe that the choice of data representation alters the predicted change in free VEGF concentrations, with the largest difference in predicted treatment efficacy given between the mode and arithmetic mean. We predict a 3.1 fold change in VEGF

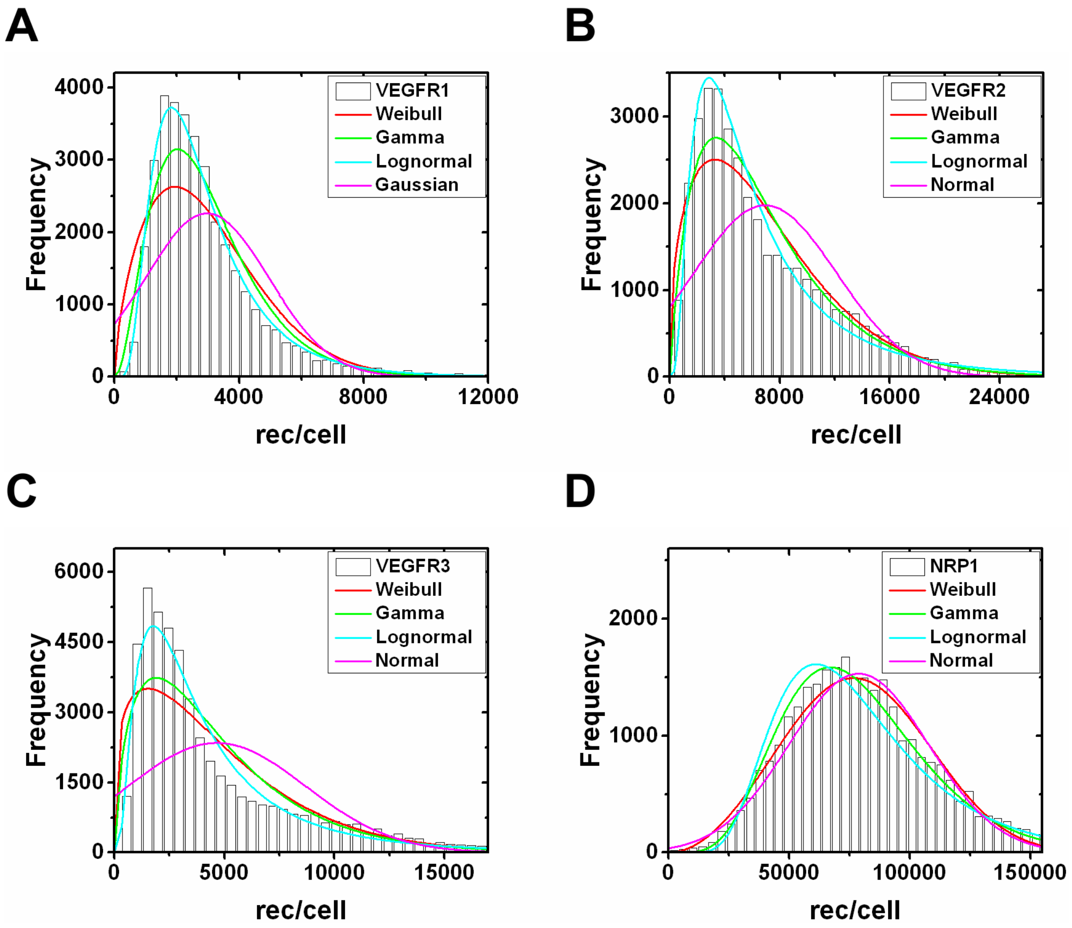

Figure 4. Statistical distribution fits to *in vitro* receptor populations. Cell-by-cell analysis of (A) VEGFR1, (B) VEGFR2, (C) VEGFR3, and (D) NRP1 distributions on *in vitro* human endothelial cells. Each distribution was fit to Weibull (generalized extreme value distribution), Gamma (maximum entropy probability distribution), and lognormal (logarithm is normally distributed) probability density functions. The parameters for the best fit distributions are given in Table 2.
doi:10.1371/journal.pone.0097271.g004

Table 2. Representative receptor levels and best fit parameters for each receptor distribution.

Receptor Type	Geometric Mean	Arithmetic Mean	Mode	Median	Weibull Parameters	Gamma Parameters	Lognormal Parameters
**H**:VEGFR1	2,530	3,000	2,720	2,500	1.67; 3,360	3.16; 940	7.83; 0.57
**H**:VEGFR2	5,260	6,950	11,400	5,350	1.43; 7,690	1.94; 3,570	8.57; 0.78
**H**:VEGFR3	3,380	4,680	1,980	3,150	1.28; 5,070	1.69; 2,750	8.13; 0.80
**H**:NRP1	74,570	81,200	87,260	77,370	2.65; 91,330	6.03; 13,460	11.22; 0.44
**C**:VEGFR1	2,100	2,970	1,820	2,050	1.24; 3,200	1.60; 1,860	7.65; 0.84
**C**:VEGFR2	1,540	2,180	2,860	1,510	1.24; 2,350	1.60; 1,360	7.34; 0.84
**B**:VEGFR1	2,700	3,850	1,700	2,650	1.20; 4,130	1.56; 2,470	7.90; 0.84
**B**:VEGFR2	1,900	2,690	1,200	1,800	1.23; 2,900	1.60; 1,680	7.55; 0.83

The number of receptors per cell given by the geometric mean, arithmetic mean, mode, and median of the data sets after applying low bin search. Data was used from HUVECs, C57BL/6 mice, and BALB/c mice, as indicated in the "Receptor Type" column by **H**, **C**, and **B**, respectively. The best fit parameters of the Weibull and Gamma distributions are given as (k; λ), and the best fit parameters of the lognormal distribution is given as (μ; σ). *In vitro* HUVEC data was quantified with flow cytometry measurements as described in [10], while the mice data was quantified with flow cytometry on primary mouse endothelial cells freshly isolated from gastrocnemius and tibialis anterior of male and female 8–10 week old mice [11].

doi:10.1371/journal.pone.0097271.t002

concentration in the blood compartment when the arithmetic mean from the C57BL/6 data is inserted into the model (2,970 VEGFR1/cell and 2,180 VEGFR2/cell) (Table 2); whereas, we predict a 1.8 fold change in the blood compartment with the mode (1,820 VEGFR1/cell and 2,860 VEGFR2/cell), and 2.1 fold change with the geometric mean (2,100 VEGFR1/cell and 1,540 VEGFR2/cell), from the same data set (C57BL/6) (Fig. 6A). The BALB/c data give similar results, predicting a 3.9 fold change in VEGF concentration in the blood compartment when updating the arithmetic mean into the model (3,850 VEGFR1/cell and 2,690 VEGFR2/cell) (Table 2); whereas, we predict a 1.6 fold change in the blood compartment with the mode (1,700 VEGFR1/cell and 1,200 VEGFR2/cell) and a 2.7 fold change with the geometric mean (2,700 VEGFR1/cell and 1,900 VEGFR2/cell) (Fig. 6B). We choose the geometric mean as the best representation of the receptor levels, and thus the most accurate fold changes, as it accounts for the tail of the distribution unlike the mode, but doesn't bias the tail like the arithmetic mean.

Free VEGF levels are sensitive to VEGFR1 levels

Since receptor levels can alter predicted anti-VEGF drug efficacy (Fig. 5, 6), we develop a healthy body model (absence of disease compartment) to better examine the receptor roles. We also add *in vitro* (Fig. 7A) or *ex vivo* (Figs. 7B-C) data in the healthy body model using all representative parameters (Table 2). The normal tissue and blood compartments give lower magnitude free VEGF levels compared to control for all simulations performed (Fig. 7). Initial free VEGF levels, relative to normalized control levels, using *in vitro* HUVEC data is notably different from the *ex vivo* C57BL/6 mouse data in the normal tissue compartment using the median (0.41 *in vitro*, 0.60 *ex vivo*) and mode (0.29 *in vitro*, 0.61 *ex vivo*). The difference between these two in the blood compartment is noticeably different using the geometric mean (0.21 *in vitro*, 0.30 *ex vivo*), median (0.14 *in vitro*, 0.33 *ex vivo*), and mode (0.06 *in vitro*, 0.32 *ex vivo*) (Fig. 7A-B). BALB/c free VEGF levels in the normal tissue compartment are notably different from those of HUVEC with the median (0.41 *in vitro*, 0.49 *ex vivo*) and mode (0.29 *in vitro*, 0.70 *ex vivo*), as well as in the blood compartment with the median (0.14 *in vitro*, 0.21 *ex vivo*) and mode (0.29 *in vitro*, 0.48 *ex vivo*) (Fig. 7A, 7C). Free VEGF concentrations versus time are also given (Fig. S2). Having added *ex vivo* VEGFRs to the normal tissue and *in vitro* and *ex vivo* VEGFRs in a tumor-free model, we compare adding *in vitro* and *ex vivo* VEGFRs to the tumor model. Free VEGF levels obtained using *ex vivo* versus *in vitro* VEGFR1 in the tumor model differ by 54% at most with the mode and by 25% at most with the geometric mean (Fig. S3).

Tumor growth increases free VEGF levels

To observe how VEGF levels may change with tumor progression, we simulate 0.62 cm^3 and 1.45 cm^3 tumor volumes, corresponding to tumors that have been growing for 3 weeks and 6 weeks, respectively. The tumor size and VEGFR1/2 surface levels on tumor cells and tumor endothelial cells (tEC) were obtained from mouse xenografts [13]. It is observed that receptor distributions on the tumor cells and tEC exhibited multiple distributions. Specifically, these tumor data contain at least 2 subpopulations arising based on size with the smaller cells containing multiple subpopulations based on receptors/cell. Due to these subpopulations, these data cannot be fit by the continuous probability distributions that we have used to fit our *in vitro* endothelial cell (HUVEC) and *ex vivo* skeletal muscle endothelial cell data. When subpopulations are qualitatively observed, fitting to mixtures is a common approach [27,28]. We have previously

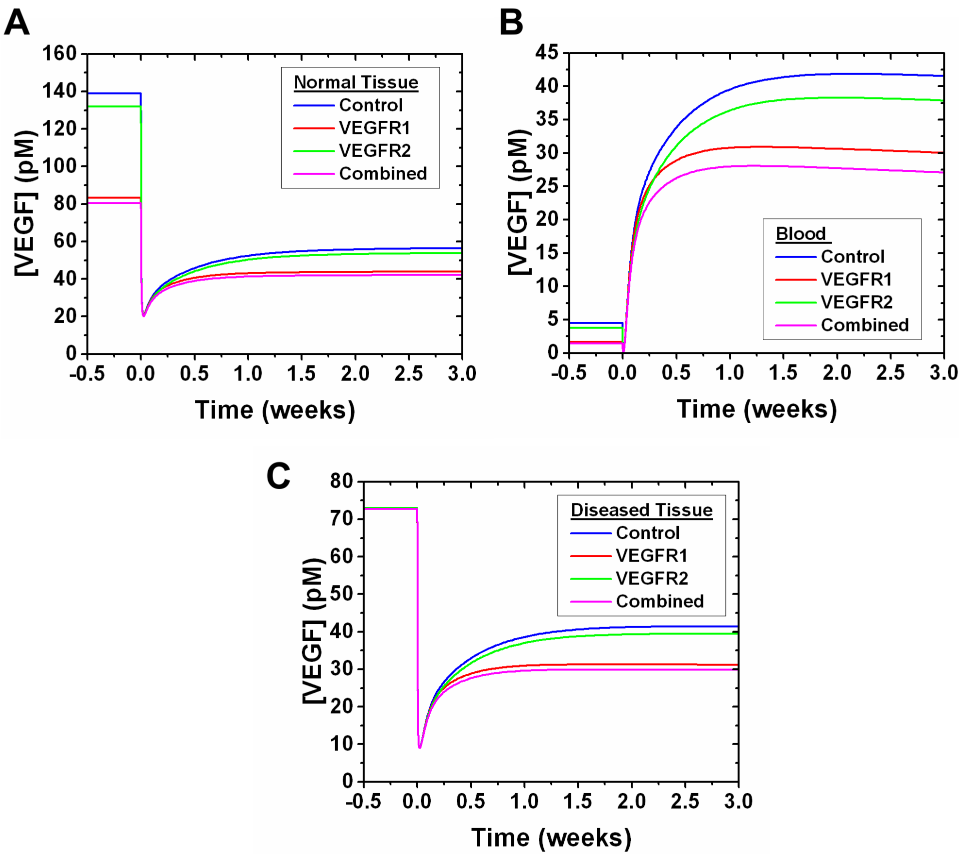

Figure 5. Effect of VEGFR1 and VEGFR2 levels on anti-VEGF efficacy. Comparison of updating the model by adding experimental VEGFR1 levels only, VEGFR2 levels only, and both compared to the control. Updated VEGFR1 and VEGFR2 values represent geometric means of C57BL/6 distributions (2,100 VEGFR1/cell and 1,540 VEGFR2/cell). The control reflects previously published VEGFR1 and VEGFR2 levels (1,100 VEGFR1/cell and 700 VEGFR2/cell) [23]. Free VEGF concentration is shown in (A) the normal tissue compartment, (B) the blood compartment, and (C) the diseased tissue compartment. An optimized anti-VEGF agent is added at t = 0 and the VEGF concentration response is simulated to 3 weeks after injection.
doi:10.1371/journal.pone.0097271.g005

modeled these populations as mixtures and shown that they fit well to the multi-component lognormal mixture model [13]. However, prior computational models have represented cellular data using the arithmetic average. Thus to arrive at a more accurate quantitative tumor cell and tEC data representation, we examine tri-modal Gaussian mixture models here. While non-Gaussian

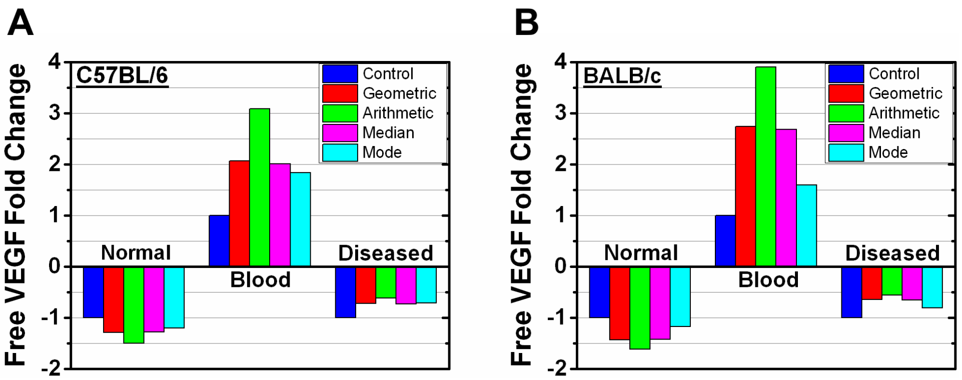

Figure 6. Effect of receptor levels on free VEGF fold change after anti-VEGF treatment. Fold change in free VEGF levels in response to anti-VEGF treatment using different representative receptor levels from the (A) C57BL/6 (C57) and (B) BALB/c (BAL) mice data. VEGFR1 and VEGFR2 levels were both updated in the model with geometric mean (2,100 VEGFR1/C57, 1,540 VEGFR2/C57, 2,700 VEGFR1/BAL, 1,900 VEGFR2/BAL), arithmetic mean (2,970 VEGFR1/C57, 2,180 VEGFR2/C57, 3,850 VEGFR1/BAL, 2,690 VEGFR2/BAL), mode (1,820 VEGFR1/C57, 2,860 VEGFR2/C57, 1,700 VEGFR1/BAL, 1,200 VEGFR2/BAL), and median (2,050 VEGFR1/C57, 1,510 VEGFR2/C57, 2,650 VEGFR1/BAL, 1,800 VEGFR2/BAL). Fold change is relative to control fold change, reflecting the normalized fold change obtained using previously published receptor levels (1,100 VEGFR1/cell and 700 VEGFR2/cell) [23].
doi:10.1371/journal.pone.0097271.g006

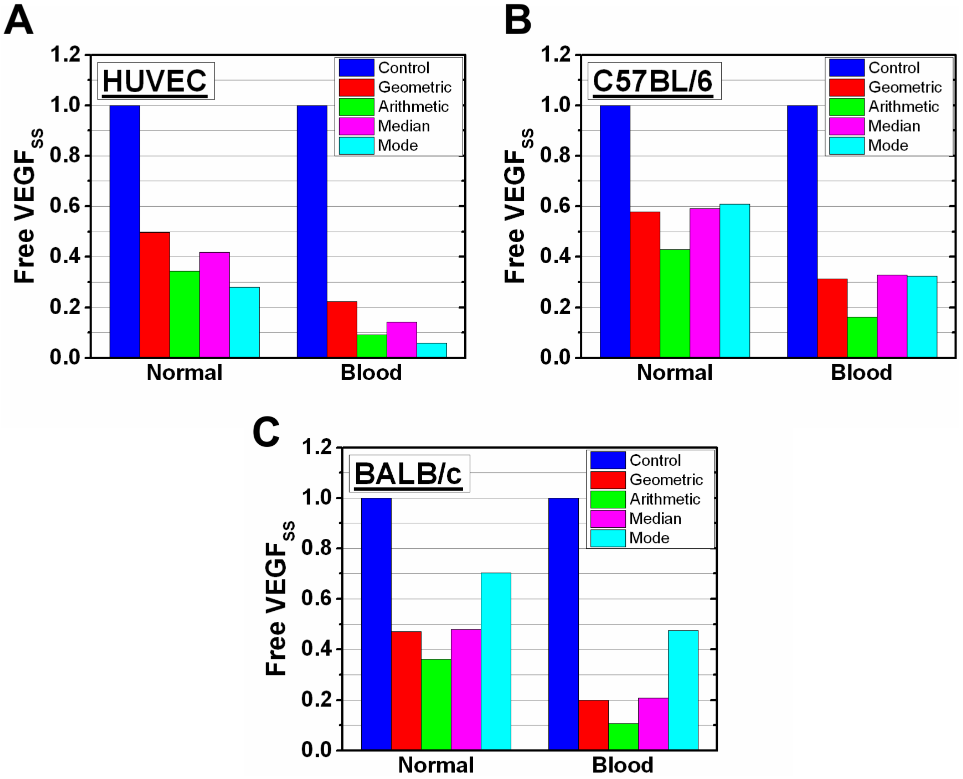

Figure 7. Receptor level effect on steady state free VEGF levels in the healthy body model. Free VEGF at steady state in the healthy body model by updating VEGFR1 and VEGFR2 levels from the (A) HUVEC, (B) C57BL/6 (C57), and (C) BALB/c (BAL) distributions. VEGFR1 and VEGFR2 levels were both updated in the model with geometric mean (2,100 VEGFR1/C57, 1,540 VEGFR2/C57, 2,700 VEGFR1/BAL, 1,900 VEGFR2/BAL, 2,530 VEGFR1/HUVEC, 5,260 VEGFR2/HUVEC), arithmetic mean (2,970 VEGFR1/C57, 2,180 VEGFR2/C57, 3,850 VEGFR1/BAL, 2,690 VEGFR2/BAL, 3,000 VEGFR1/HUVEC, 6,950 VEGFR2/HUVEC), mode (1,820 VEGFR1/C57, 2,860 VEGFR2/C57, 1,700 VEGFR1/BAL, 1,200 VEGFR2/BAL, 2,720 VEGFR1/HUVEC, 11,400 VEGFR2/HUVEC), and median (2,050 VEGFR1/C57, 1,510 VEGFR2/C57, 2,650 VEGFR1/BAL, 1,800 VEGFR2/BAL, 2,500 VEGFR1/HUVEC, 5,350 VEGFR2/HUVEC). The control reflects previously published VEGFR1 and VEGFR2 levels (1,100 VEGFR1/cell and 700 VEGFR2/cell) [23].
doi:10.1371/journal.pone.0097271.g007

mixture models, such as the Weibull mixture model [29], have more flexibility in shape than the Gaussian mixture model, due to both the goodness of fit and simplicity in computational resources, we believe the Gaussian mixture model is the best model choice, and deem it unnecessary to perform additional fitting to other mixture models. Simulations are performed (1) using the tri-modal mixture distribution and (2) individually inserting each component (mean) comprising the tri-modal mixture. Receptor levels within the mixture model are determined by weighting the individual Gaussian distribution parameters with their respective densities and summing. The distribution and mixture fits to receptor levels on tumor cells and tECs grown for 3 weeks and 6 weeks are shown (Fig. 8–9). The densities are ordered with "Density 1" having the highest cell frequency and "Density 3" having the lowest frequency. As the tumor grows from 3 weeks to 6 weeks, VEGFR1 subpopulations on the tumor cells are more highly expressed (Fig. 8A, 9A), but VEGFR2 levels only slightly change (Fig. 8B, 9B). For example, "Density 1" gives the geometric mean at 2,900 VEGFR2/tumor cell (Table 3) at week 3 (Fig. 8B), and "Density 1" gives the geometric mean at 3,150 VEGFR1/tumor cell (Table 3) at week 6 (Fig. 9B). VEGFR1/2 levels on tECs decrease throughout tumor growth. For example, "Density 1" gives the geometric mean at 13,000 VEGFR1/tEC (Table 3) at week 3 (Fig. 8C), and "Density 1" gives the geometric mean at 600 VEGFR1/tEC (Table 3) at week 6 (Fig. 9C). Likewise, "Density 1" gives the geometric mean at 1,450 VEGFR2/tEC (Table 3) at week 3 (Fig. 8D), and "Density 1" gives the geometric mean at 600 VEGFR2/tEC (Table 3) at week 6 (Fig. 9D). Gaussian distributions corresponding to receptor subpopulations express large differences in receptor levels (Table 3).

At tumors grown to 3 weeks, there are no noticeable changes in free VEGF levels in either the normal or the blood compartments when only the tEC receptor levels are updated, for all receptor distributions (Fig. 10A-B, 10D-E, 10G-H). However, initial free VEGF levels before anti-VEGF administration in the diseased tissue compartment is highly dependent on the VEGFR1 levels on tECs (Fig. 10C, 10F, 10I). Free VEGF levels in the diseased tissue converge to approximately 41 pM after anti-VEGF treatment.

Interestingly, using the VEGFR1 geometric mean on the "Density 2" distribution (Fig. 8C), which is 69,500 VEGFR1/cell (Table 3), gives an increase in free VEGF levels in the diseased tissue after anti-VEGF treatment, corresponding to a 1.64 fold change in free VEGF (Fig. 10C). Sensitivity analysis, performed by observing free VEGF levels in response to parameter perturbations, reveals that decreasing the VEGFR1 membrane insertion rate on the tECs transforms anti-VEGF treatment from a pro-angiogenic response to anti-angiogenic (Fig. 11). The insertion rate defines the rate at which receptors are inserted into the cell membrane, such as through receptor trafficking, making those receptors available for ligand binding. Sensitivity analysis also reveals that anti-VEGF treatment was ineffective for subpopulations containing more than 35,000 VEGFR1/cell (data not shown).

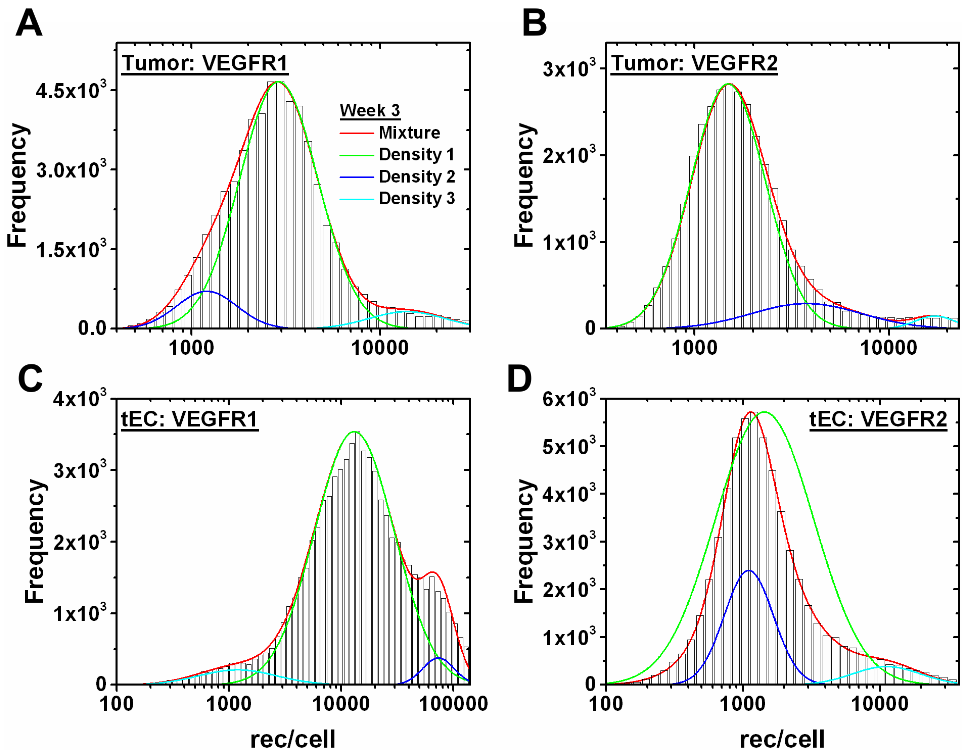

Figure 8. Gaussian mixture model of tECs and tumor cells at 3 weeks of tumor growth. Gaussian tri-modal mixture models and the individual Gaussian distributions making up the mixture model for (A) VEGFR1 on tumor cells, (B) VEGFR2 on tumor cells, (C) VEGFR1 on tECs, and (D) VEGFR2 on tECs at 3 weeks of tumor growth. "Density 1" corresponds to the Gaussian with highest weight in the mixture model, "Density 2" is the second highest weight, and "Density 3" is the lowest weight.
doi:10.1371/journal.pone.0097271.g008

As with tumors grown to 3 weeks, tumor grown to 6 weeks show no noticeable change in free VEGF levels in the normal and blood compartments when only the tEC receptor levels were updated (data not shown). Initial free VEGF levels in the diseased tissue compartment, before adding anti-VEGF, are dependent on tEC receptor levels, but converge to approximately 41 pM after administering anti-VEGF (Fig. 12A-C). Free VEGF levels in the diseased tissue compartment before anti-VEGF treatment is higher in the 6 week tumor than in the 3 week tumor. For example, using the geometric mean and updating both tEC receptors, the mixture model of the 6 week tumor gave a free VEGF concentration of 93.77 pM (Fig. 12C) while the 3 week tumor gave 49.24 pM (Fig. 10I). The tumor cell receptors are updated in a similar fashion for 3 week (Fig. S4) and 6 week tumors (Fig. S5). Updating VEGFR1/2 levels on the tumor cells either individually or simultaneously gave no noticeable change to the free VEGF levels in the normal tissue and blood compartments for all receptor distributions, but free VEGF levels are highly sensitive to the tumor cell receptor levels in the diseased tissue (Fig. S4, S5).

Remark. The receptor levels and tumor sizes are obtained using mouse xenograft models: human tumor inoculated in mouse; therefore, the tumor cells are human and the tumor endothelial cells are murine. However, all values (mouse and human) are used in simulating an adult human. This simplification is acceptable as we are only interested in observing trends in the free VEGF levels in response to receptor heterogeneity. These observed trends are still physiologically relevant for humans as we assume the tumor size ratio to receptor levels are the same for adult humans.

Discussion

In this study, we introduce a novel method for quantitatively representing heterogeneous populations, and show how accounting for heterogeneity affects drug efficacy predictions. We show how low bin search simultaneously define outliers and optimizes heterogeneous data binning. This is accomplished by performing goodness of fit tests to various statistical distributions, where the best fitting statistical distribution is determined. The best fitting statistical distribution is then used to define outliers and then representative parameters from the data are extracted. We used these parameters in a tumor angiogenesis model, where we predict that free VEGF levels are sensitive to VEGFR1. Using the simplified healthy body model, we also observed that free VEGF levels are sensitive to VEGFR1 expression, indicating that cellular heterogeneity is essential in both healthy and cancerous angiogenesis models. We also predict that anti-VEGF drug efficacy is sensitive to subpopulations present in tumor cells and tECs.

Defining outliers in heterogeneous populations

We introduced low bin search, a novel method to define outliers in a heterogeneous distribution that follows the algorithm: (1) determining the best fitting distribution with the minimum SSE; (2) finding the optimal bin number by minimizing the SSE the best fit distribution gives over a range of histograms; (3) defining the cutoff point from the optimal bin number. The cutoff point eliminates the first bin that meets two criteria: (1) the number of cells in the cutoff bin is less than 1% the number of cells in the largest bin; (2) the neighboring bins also have a number of cells less than 1% the number of cells in the largest bin. The theoretical

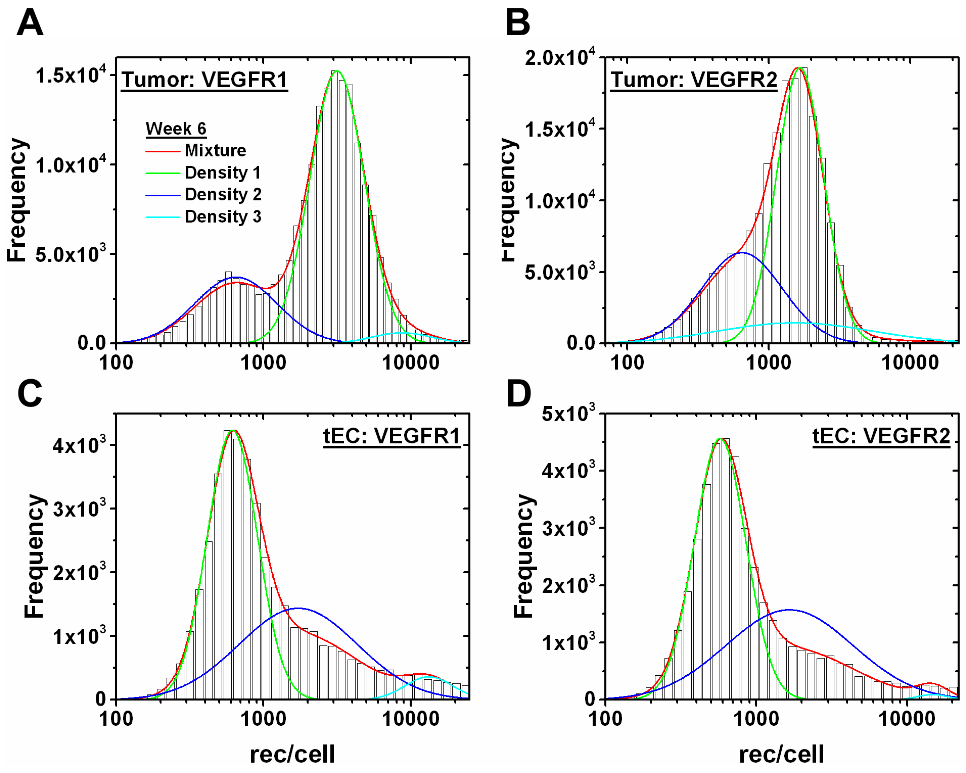

Figure 9. Gaussian mixture model of tECs and tumor cells at 6 weeks of tumor growth. Gaussian tri-modal mixture models and the individual Gaussian distributions making up the mixture model for (A) VEGFR1 on tumor cells, (B) VEGFR2 on tumor cells, (C) VEGFR1 on tECs, and (D) VEGFR2 on tECs at 6 weeks of tumor growth. "Density 1" corresponds to the Gaussian with highest weight in the mixture model, "Density 2" is the second highest weight, and "Density 3" is the lowest weight.
doi:10.1371/journal.pone.0097271.g009

basis is as follows: the first criterion ensures the cutoff bin has low occurrence probability, while the second criterion ensures uniqueness. Thus, as low bin search is an algorithmic approach for defining outliers with a theoretical basis for defining the cutoff point, we define it as a method as opposed to a heuristic rule, which pertains to trial-and-error methods of problem solving when an algorithmic approach is infeasible or a theoretical basis is lacking. This novel method represents an important development, since outlier detection is essential for removing unwanted data or finding features that occur at low probability. Outlier detection has been tackled for mass spectrometry by calculating the Mahalanobis distance a distribution gives to all other experimental runs and eliminating those with suspiciously high distances [30]. Another algorithm uses projection and quantile regression to discard values that do not follow the general trend given by the data set [31], but is computationally intensive for many data points. These methods are useful for removing experimental runs that exhibit high variation, but not for defining outliers within a data set. Outliers in gene expression can be defined by incorporating probability distribution functions and comparing to multiple experimental runs [32]. However, this method for gene expression requires *a priori* knowledge for population distribution functions, which may not be available. The low bin search method we present requires no *a priori* knowledge in regards to what statistical distribution represents the data, and will define outliers within any type of distribution.

Automatic gating and outlier removal in flow cytometry

Flow cytometry is a high-throughput tool for cell-by-cell analysis, and requires gating to separate cell populations. Bashashati and Brinkman have emphasized the need to completely automate flow cytometry data analysis, and provided a theoretical framework for this analysis [33]. However, few methods for automatically gating flow cytometry data and defining outliers within gated data exist. One such method for automated gating is cluster analysis, which groups cells with similar fluorescence patterns, such as by K-means clustering [34,35]. Density based merging places the flow cytometry data on a 2-dimensional grid, and groups data based on grid point densities [36]. K-means cluster is limited in that clusters must be convex, and thus neglect subpopulation features exhibited by any other shape. Density based merging is limited to gating 2-dimensional data, such as data containing forward scatter and side scatter information only, and thus cannot gate data with more than two features. There is a need to expand on current methods to gate high dimensional data and better capture subpopulations. Thus, due to current automated gating limitations, gating typically involves manually selecting cell populations for these data as we have done in the past [10–13]. The low bin search method we developed could be implemented in addition to automatic gating to search through and remove outliers within a gate. This would provide less manual pre-processing of flow cytometry data, which is time consuming and subject to human error and reproducibility issues.

Binning optimization

Low bin search presented here bins data based on the data shape and does not require transforming statistical distributions. Binning is important as it reduces data dimensionality and organizes data based on similar features. Mathematic operations performed on binned data, such as fitting statistical distributions,

Table 3. Representative receptor levels obtained from each tri-modal Gaussian mixture model.

Whole Cell Values	Week 3 tEC R1	tEC R2	Tumor R1	Tumor R2	Week 6 tEC R1	tEC R2	Tumor R1	Tumor R2
**M**:Geometric	18,550	1,950	3,300	2,200	1,500	1,100	2,800	1,250
**M**:Mode	19,450	2,000	3,400	2,300	1,500	1,100	2,850	1,300
**D1**:Geometric	13,000	1,450	2,900	1,500	600	600	3,150	950
**D1**:Mode	13,700	1,400	3,000	1,500	600	650	3,300	1,000
**D2**:Geometric	69,500	1,100	1,200	3,750	1,700	2,400	650	1,500
**D2**:Mode	72,450	1,050	1,250	3,950	1,600	2,300	650	1,500
**D3**:Geometric	1,200	10,900	13,250	14,950	12,250	600	8,500	2,300
**D3**:Mode	1,150	11,000	13,500	16,800	13,150	600	8,550	2,300

The geometric mean, arithmetic mean, and mode of the mixture model and the three Gaussian distributions that make up the mixture model for all cell types. "Density 1", **D1**, corresponds to the Gaussian with highest weight in the mixture model **M**, whereas "Density 2", **D2**, is the second highest weight and "Density 3", **D3**, is the lowest weight. The mixture was obtained by summing the geometric means of each density distribution weighted by their density in the mixture model. Tumor cell and tEC receptor levels were quantified using flow cytometry on MDA-MB-231 xenografts, as previously described [13].
doi:10.1371/journal.pone.0097271.t003

are affected by the binning method. Thus, it is important to optimize the number of bins such that statistical distributions can best represent the data. Current approaches are limiting because they arbitrarily assign a number of bins to represent the data based solely on the number of data points. For instance, two commonly used software packages, MATLAB and Palisade, determine the number of bins based on $\sqrt{n}$ and rounding up to the nearest integer, or by $10\log(n)$ and rounding down to the nearest integer, for n data points, respectively. To improve these current approaches, it is necessary to develop methods that bin based on specific data features, in order to best represent each data set individually. Thus, we created low bin search to bin and represent data sets that optimizes the fit to the best fitting statistical distribution. For comparison, low bin search determined that NRP1 levels on HUVECs are optimally distributed over 101 bins, whereas MATLAB would use 172 bins and Palisade would use 44 bins. Using these bin numbers, the total error was calculated as the sum of the individual SSE from the Weibull, Gamma, and lognormal fits. For this example, MATLAB gave a total error 24.71% larger than that given by low bin search, while Palisade gave a total error 77.41% larger than low bin search. Probability Binning is another binning method, which splits up the data such that every bin may not have the same width, but contains an equal number of events [37]. However, this method shapes the histogram into a uniform distribution, and requires transforming statistical probability distributions for goodness of fit tests. Low bin search not only effectively defines outliers, but also optimizes data binning.

Determining the best fitting statistical distributions

We show that it is necessary to determine the goodness of fit a statistical distribution gives to a heterogeneous data set to find the best fitting distribution. There are many metrics for testing the goodness of fit between observed data and statistical distributions. We chose SSE as the test metric as it weights every bin equally. By changing the number of bins, the cluster mean changes without altering the best fitting distribution, allowing the SSE using different bin numbers to be compared. We optimize the bin number with the minimum SSE, and the outliers are then defined. Thus, using SSE to measure goodness of fits not only determines the best fit distribution, but simultaneously defines outliers. For binned data, SSE compares the observed hits in a bin to that expected from a statistical distribution, using the bin center. Another test metric, Chi-squared, differs as it weights each bin by the number of hits it contains [38]. Overfitting is a significant problem that negates predictive power, and results in bad data fitting. Overfitting occurs when a statistical model is overly complex to explain the data, typically describing random noise or errors instead. Overfitting generally occurs when there are a relatively high number of fit parameters to the number of observations. To prevent overfitting, low bin search performs the following: (1) finds the best fitting distribution; for example, in the case of VEGFR3 this best fitting distribution is the lognormal distribution (Fig. 4C); (2) optimizes the number of bins by minimizing the SSE given by the best fitting distribution (Eqn 5); (3) determines the cutoff point (Eqn 7). The optimal bin number is found by minimizing the SSE the best fitting distribution gives, as opposed to minimizing the SEE globally for all distributions, reducing the overfitting risk. Additionally, all data sets contain greater than 20,000 observations, but rather than attempting to fit all observations, we fit to data represented by the optimal bin number. This further reduces fitting parameters and the overfitting risk. Further developing low bin search could include comparing goodness of fit tests to determine the most robust test metric.

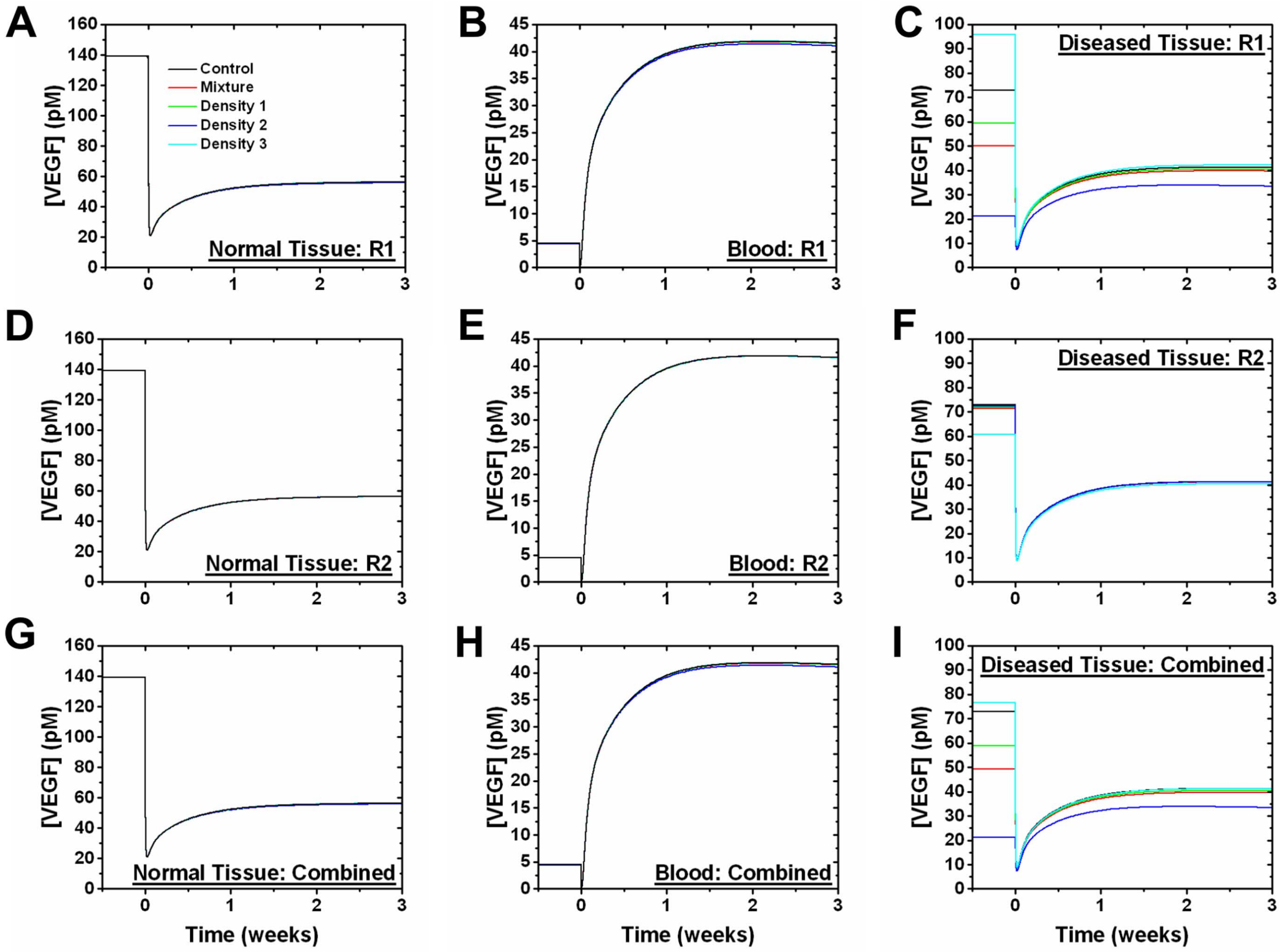

Figure 10. Effect of tEC receptor levels on anti-VEGF treatment at 3 weeks of tumor growth. Free VEGF in the normal tissue, blood, and diseased tissue compartments in response to anti-VEGF treatment after updating (A-C) VEGFR1 alone, (D-F) VEGFR2 alone, and (G-I) both receptors on the tECs at 3 weeks of tumor growth. "Density 1" (D1) corresponds to the Gaussian with highest weight in the mixture model, whereas "Density 2" (D2) is the second highest weight and "Density 3" (D3) is the lowest weight. "Mixture" was obtained by summing the geometric means of each density distribution weighted by their density in the mixture model. The geometric mean was used for all receptor distributions (18,550 VEGFR1/Mixture, 1,950 VEGFR2/Mixture, 13,000 VEGFR1/D1, 1,450 VEGFR2/D1, 69,500 VEGFR1/D2, 1,100 VEGFR2/D2, 1,200 VEGFR1/D3, 10,900 VEGFR2/D3). The control reflects previously published VEGFR1 and VEGFR2 levels (1,100 VEGFR1/tEC and 700 VEGFR2/tEC) [23].
doi:10.1371/journal.pone.0097271.g010

Statistical distributions best fitting data sets

We have found that VEGFRs best fit to lognormal distributions under all experimental conditions, whereas NRP1 best fit to the Gamma distribution when untreated and to the Weibull distribution when treated with VEGF-A or VEGF-C. We hypothesize VEGFRs best fit lognormal distributions because they have heavy tails not present in NRP1 distributions. The heavy tail shows that some cells in the population express receptors at a higher level, which may be used by the cell population to elicit a specific response. Lacking a heavy tail in the NRP1 distributions indicates that the cell population keeps NRP1 levels relatively consistent across the population. Previous studies have shown that NRP1 expression is important for T-cell stability and survival, and may have a similar role in endothelial cells [39]. Additionally, NRP1 is a co-receptor for VEGF-A_{165}, and appears to play a vital role in vascular morphogenesis [40]. However, it is not known why NRP1 is expressed at higher levels than VEGFRs. Additionally, better understanding the connection between the best fitting statistical distribution and the population role is needed.

Quantifying a heterogeneous population

Due to the VEGFRs being best fit to lognormal distributions, we chose the geometric mean as the best heterogeneous data representation. This is because the geometric mean accounts for the heavy tail, unlike the mode or median, but it is not biased towards the tail like the arithmetic mean. We do not want to bias the heavy tail as it only represents a small subset of the cells in the population. For example, VEGFR1 on tECs at 3 weeks had a heavy tail that accounted for approximately 3% the total cells in the population. The three statistical distributions were chosen due to their characteristic properties; Weibull is a special case of the generalized extreme value distribution which approximates the maxima of a finite sequence of random variables; Gamma is the maximum entropy probability distribution which chooses the unknown distribution that exhibits the highest entropy; and lognormal fits a distribution whose logarithm follows a Gaussian. Representative parameters were chosen as they are the best parameters that represent the three statistical distributions; they are calculated the same for each distribution without biasing one

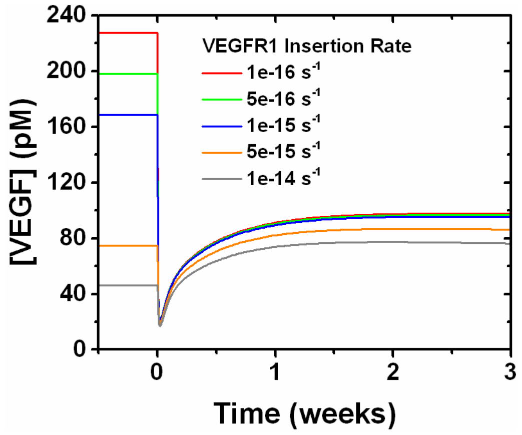

Figure 11. The insertion rate of VEGFR1 tunes the anti-angiogenic effect of anti-VEGF treatment. Sensitivity analysis of the insertion rate of VEGFR1 into the tEC membrane tunes the efficacy of anti-VEGF treatment. This example examines "Density 2" at 3 weeks of tumor growth giving 69,500 VEGFR1/tEC. The insertion rate using 69,500 VEGFR1/tEC is approximately 1e-14 s^{-1}, where anti-VEGF treatment provides a pro-angiogenic response. Decreasing the insertion rate allows for anti-VEGF treatment to provide an anti-angiogenic response.
doi:10.1371/journal.pone.0097271.g011

distribution over the other. These representation parameters are thus generalized for any heterogeneous data set and require no *a priori* knowledge. Expanding low bin search to include more statistical distributions will allow for more accurate heterogeneous data representations.

VEGFR1 levels control free VEGF levels

Free VEGF levels are more sensitive to VEGFR1 than VEGFR2: using the C57BL/6 geometric mean from healthy endothelial cells, we observed that updated VEGFR1 alone resulted in a steady state free VEGF concentration change of 24.6% compared to control in the diseased tissue, while VEGFR2 alone gave a 4.8% change. As VEGFR1 and VEGFR2 on the healthy endothelial cells are altered, we would expect free VEGF levels in the healthy tissue and blood compartments to change as those compartments are where the healthy endothelial cell receptors are located. Previous studies confirm that free VEGF concentrations are more sensitive to VEGFR1 than VEGFR2 levels, due to a higher binding affinity to VEGFR1 [10]. As such, VEGFR1 is typically identified as a decoy receptor [41]. However, more insight is necessary into the complete function of VEGFR1.

VEGFR1 is important for cell migration

We have shown a high VEGF binding affinity and sensitivity to VEGFR1, indicating that VEGFR1 plays a more important role than simply being a decoy. For HUVECS, VEGFR1 mediates p38 phosphorylation, which controls cell migration through actin reorganization, whereas VEGFR2 mediates ERK1/2 phosphorylation, which mediates cell proliferation [42,43]. VEGFR1 is expressed at high levels on BALB/c fibroblasts (36,000 rec/cell) [11], and fibroblast migration can be abolished with bevacizumab [44]. This VEGFR1 migratory function is also observed in macrophages [45,46], monocytes [47–49], and endothelial cells [50]. If the VEGFR1 migratory function observed in these studies also translates to the tumor endothelial cells, this would explain the high VEGF binding to VEGFR1 observed in our computational model.

Several studies have also suggested a role for upregulated VEGFR1 in migration. In murine hindlimb ischemia, VEGFR1, as well as proliferation markers and migration, are upregulated on ischemic endothelial cells ten days post ischemic induction; this time point coincides with accelerated perfusion recovery to the hindlimb [12]. Another study has shown that VEGFR1 is expressed 3 days after embyroid body induction, a time marked by significant migratory phenotype by endothelial PmTc1 cell lines. VEGFR1 expression is sustained through day ten, at which point the endothelial-like cells arrange in a distinct pattern to line vessel-like structures [51]. Gastric ulcers were induced in RAG2 mice, and gastric ulcer healing in VEGFR1 knockout mice resulted in decreased CD31 mRNA levels and decreased microvessel density compared to wildtype mice, indicating that VEGFR1 has an important role in cell migration and vessel formation in gastric ulcer healing [52]. Additionally, VEGFR1 expression on highly metastatic 3LL-LLC tumor cells induces MMP9 expression in premetastatic lung endothelial cells and macrophages. MMP9 then breaks down the extracellular matrix and allows tumor cell migration [53]. It should be noted that

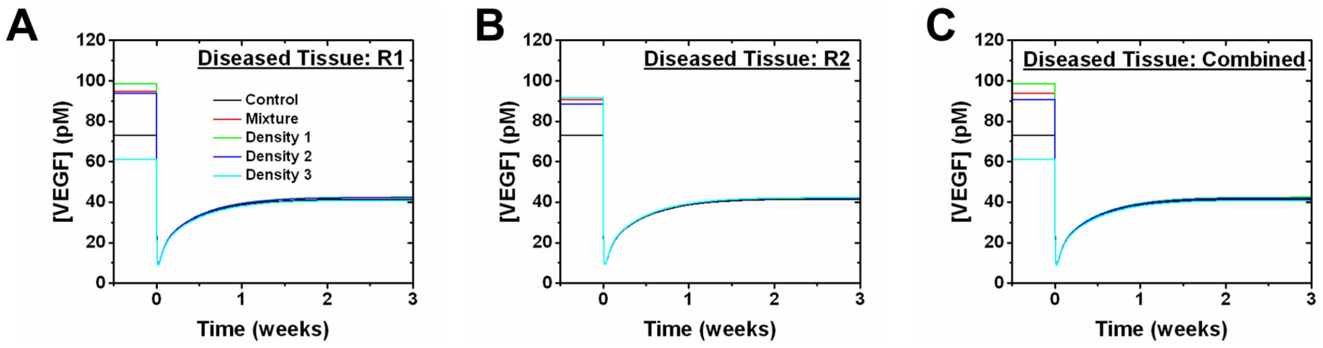

Figure 12. Effect of tEC receptor levels on anti-VEGF treatment at 6 weeks of tumor growth. Free VEGF in the diseased tissue compartment in response to anti-VEGF treatment after updating (A) VEGFR1 alone, (B) VEGFR2 alone, and (C) both receptors on the tECs at 6 weeks of tumor growth. "Density 1" (D1) corresponds to the Gaussian with highest weight in the mixture model, "Density 2" (D2) is the second highest weight and "Density 3" (D3) is the lowest weight. "Mixture" was obtained by summing the geometric means of each density distribution weighted by their density in the mixture model. The geometric mean was used for all receptor distributions (1,500 VEGFR1/Mixture, 1,100 VEGFR2/Mixture, 600 VEGFR1/D1, 600 VEGFR2/D1, 1,700 VEGFR1/D2, 2,400 VEGFR2/D2, 12,250 VEGFR1/D3, 600 VEGFR2/D3). The control reflects previously published VEGFR1 and VEGFR2 levels (1,100 VEGFR1/tEC and 700 VEGFR2/tEC) [23].
doi:10.1371/journal.pone.0097271.g012

VEGFR1 does not appear to modulate neuronal migration – lowly expressed VEGFR1 on cortical neuron cells was blocked with a polyclonal antibody and neurogenesis was not hindered [54]. Likewise, neurons expressed no VEGFR1 mRNA when the cells were treated with VEGF-A_{165}, indicating VEGFR1 is unimportant for neurogenesis [55]. Altogether, our results, and these previous studies, support the idea that VEGFR1 is upregulated at early angiogenesis stages and is a crucial regulator of cell migration.

The healthy body model assures physiological relevancy

For systems biology studies, building and testing model modules assures that individual model pieces give physiologically relevant results before adding additional modules. The healthy body model was used, as it allows the heterogeneity effects to be observed in a simplified model. This allowed us to sequentially insert experimentally obtained receptor levels on healthy endothelial cells and observe the effect in the healthy tissue compartment they reside in, without any cross talk from the tumor. Initial model versions contained a single, normal tissue compartment, and provided insight into the importance in VEGFR levels in determining VEGF concentration [56]. This iterative process allows individual molecules or parameters to be understood, as well as provides a troubleshooting basis for more complex models.

Tumor associated cells control drug efficacy

VEGFR1/2 levels on tumor cells and tECs play an important role in determining drug efficacy. By observing free VEGF levels in the diseased tissue compartment, we have shown that anti-VEGF treatment is ineffective against specific tumor cell and tEC subpopulations that have high expression levels. These high expression levels consequently increased the membrane protein insertion rate, and we showed that decreasing the insertion rate recovered the anti-angiogenic result from anti-VEGF treatment. For these subpopulations, receptor mRNA could be targeted to reduce receptor levels or the insertion rate could be decreased by inhibiting insertion proteins [57]. tECs can also form various vessel types, creating a highly heterogeneous tumor vasculature [58]. It is possible the formed blood vessel type varies with time, with early vessels being more responsive to anti-VEGF treatment and late vessels portraying anti-VEGF resistance [59]. The hierarchy of large arteries bifurcating into successively smaller conduits present in normal blood vessel networks is missing in tumor vasculature, which often lack sufficient blood flow, pericyte coverage, and exhibit leakiness and dilation [60,61]. These changes in tumor vasculature could account for the heterogeneous subpopulations that we observe in tECs. One study aiming to characterize cell subpopulation heterogeneity decomposed populations based on basal signaling markers and showed cells with similar basal signaling to have similar drug sensitivities [62], while others have characterized subpopulations through signaling markers after applying drug treatment [63]. Singh *et al* noted that characterizing subpopulations in an ensemble manner may be required to distinguish biological or functional differences in subpopulations. These tEC subpopulations and heterogeneities in vessel formation and properties indicate a need to better characterize cell subpopulations and their potential responses to drug treatment. Our work provides insight into subpopulation dispersion with regards to VEGFR membrane localization and how this affects drug treatment.

Incorporating cellular heterogeneity with signaling pathways

We present here how receptor heterogeneity affects ligand binding kinetics and ligand distributions. The model implemented here is a simplified angiogenesis model as it does not account for receptor trafficking or cell signaling pathways. However, computational studies require building models in an iterative manner to ensure physiological relevancy, and the lack of previous studies computationally exploring cellular heterogeneity necessitates this simplified model. For instance, early computational models sought to reveal the mechanism of EGFR activation via EGF binding using simplified models to examine the interaction kinetics [64,65]. Later models then sought to better understand the cellular complexity pertaining to EGFR activation by mapping the downstream signaling and receptor internalization [19,66,67]. However, VEGF models have not yet reached the sophistication that EGF models have [68], highlighting the necessity to examine VEGF model sensitivity. Prior sensitivity analysis revealed that VEGFRs are the primary controller of free VEGF levels [56]. Early VEGF models first examined ligand binding kinetics to VEGFR [69] or receptor dimerization kinetics [70] in simplified models. Later studies increased complexity by modeling tumor angiogenesis to examine the effect of the tumor microenvironment on anti-VEGF treatment efficacy [71,72], and included targeting specific VEGF isoforms [73]. Recent models incorporated receptor trafficking and specific signaling pathways to better understand cellular activity upon ligand binding [41,68] using ensemble averaged receptor levels. Therefore, accurate VEGFR representations are necessary for accurate model development. Our ability to obtain these experimental data on a cell-by-cell level allowed us to now examine the most appropriate way to analyze the data, represent the data, and extract representative parameter(s) for use in models. Here, we show that these are critical steps for accurate model development. We similarly show how data representation affects the ligand binding kinetics and ligand distribution in a simplified model. We use free VEGF levels as the functional model output, since it is the primary signaling molecule in angiogenesis [74], its secretion by tumor mediates tumor growth and metastasis [75], and a meta-analysis has shown that VEGF levels in blood and serum is significantly elevated in cancer patients [76]. We believe that our work presents a novel foundation for understanding heterogeneity, particularly in tumor, and with such a foundation, we can now incorporate downstream signaling for more reliable predictive power.

Concluding remarks

Here we show how the choice of data representation in heterogeneous populations can affect anti-angiogenic drug efficacy predictions. While we select a single value from the populations to represent the heterogeneity (every cell in the population contains that representative parameter), we present significant analysis to support the parameter representation; thereby establishing the need to fully analyze experimental data and comprehensively identify the effect of data representation on model predictions. With the advent of high-throughput cell-by-cell data, compartments should incorporate these cell-by-cell data as individual cells with simulations probing the aggregation of these cell-by-cell dynamics. Such approaches, applied to our system, would require expanding the single normal tissue compartment to $h+1$ compartments for h healthy endothelial cells, expanding the diseased tissue compartment to $t+d+1$ compartments for d tECs and t tumor cells, and expanding the blood compartment to $h+t+1$ compartments. Even for relatively low numbers of cells, say 100,000 of each type, we estimate a single simulation could

take upwards of 170 hours/7 days on the system used for this study. This increased complexity would require supercomputing, split among multiple processors to obtain reasonable solution times. Extensions such as these would advance new insight into how a system of distinct cells interacts to achieve a specific response.

Supporting Information

Figure S1 Statistical distribution fits to *ex vivo* receptor populations. Cell-by-cell analysis of VEGFR1/2 distributions from C57BL/6 (A-B) and BALB/c (C-D) mice. Each distribution was fit to Weibull (generalized extreme value distribution), Gamma (maximum entropy probability distribution), and lognormal (logarithm is normally distributed) probability density functions. The parameters for the best fit distributions are given in Table 2.
(TIF)

Figure S2 Receptor level effect on free VEGF levels in the healthy body model. Comparison of adding *ex vivo* or *in vitro* receptor levels in the healthy body model. VEGF concentration was initialized at 0 pM and simulated to steady state. The geometric mean (2,100 VEGFR1/C57, 1,540 VEGFR2/C57, 2,700 VEGFR1/BAL, 1,900 VEGFR2/BAL, 2,530 VEGFR1/HUVEC, 5,260 VEGFR2/HUVEC) and mode (1,820 VEGFR1/C57, 2,860 VEGFR2/C57, 1,700 VEGFR1/BAL, 1,200 VEGFR2/BAL, 2,720 VEGFR1/HUVEC, 11,400 VEGFR2/HUVEC) of the distributions were used. (A,D) Free VEGF response in updating VEGFR1 alone, (B,E) updating VEGFR2 alone, and (C,F) updating both simultaneously. The control reflects previously published VEGFR1 and VEGFR2 levels (1,100 VEGFR1/cell and 700 VEGFR2/cell) [23].
(TIF)

Figure S3 Receptor level effect on free VEGF levels in the tumor model. Comparison of adding *ex vivo* or *in vitro* receptor levels in the tumor model. An optimized anti-VEGF agent is added at $t = 0$ and the VEGF concentration response is simulated to 3 weeks after injection. The geometric mean (2,100 VEGFR1/C57, 1,540 VEGFR2/C57, 2,700 VEGFR1/BAL, 1,900 VEGFR2/BAL, 2,530 VEGFR1/HUVEC, 5,260 VEGFR2/HUVEC) and mode (1,820 VEGFR1/C57, 2,860 VEGFR2/C57, 1,700 VEGFR1/BAL, 1,200 VEGFR2/BAL, 2,720 VEGFR1/HUVEC, 11,400 VEGFR2/HUVEC) of the distributions were used. (A-C) Free VEGF response in updating VEGFR1 alone, (D-F) updating VEGFR2 alone, and (G-I) updating both simultaneously. The control reflects previously published VEGFR1 and VEGFR2 levels (1,100 VEGFR1/cell and 700 VEGFR2/cell) [23].
(TIF)

Figure S4 Effect of tumor cell receptor levels on anti-VEGF treatment at 3 weeks of tumor growth. Free VEGF in the normal tissue, blood, and diseased tissue compartments in response to anti-VEGF treatment after updating (A-C) VEGFR1 alone, (D-F) VEGFR2 alone, and (G-I) both receptors on the tumor cells at 3 weeks of tumor growth. "Density 1" (D1) corresponds to the Gaussian with highest weight in the mixture model, "Density 2" (D2) is the second highest weight, and "Density 3" (D3) is the lowest weight. "Mixture" was obtained by summing the geometric means of each density distribution weighted by their density in the mixture model. The geometric mean was used for all receptor distributions (3,300 VEGFR1/Mixture, 2,200 VEGFR2/Mixture, 2,900 VEGFR1/D1, 1,500 VEGFR2/D1, 1,200 VEGFR1/D2, 3,750 VEGFR2/D2, 13,250 VEGFR1/D3, 14,950 VEGFR2/D3). The control reflects previously published VEGFR1 and VEGFR2 levels (1,100 VEGFR1/cell and 700 VEGFR2/cell) [23].
(TIF)

Figure S5 Effect of tumor cell receptor levels on anti-VEGF treatment at 6 weeks of tumor growth. Free VEGF in the normal tissue, blood, and diseased tissue compartments in response to anti-VEGF treatment after updating (A-C) VEGFR1 alone, (D-F) VEGFR2 alone, and (G-I) both receptors on the tumor cells at 6 weeks of tumor growth. "Density 1" (D1) corresponds to the Gaussian with highest weight in the mixture model, "Density 2" (D2) is the second highest weight, and "Density 3" (D3) is the lowest weight. "Mixture" was obtained by summing the geometric means of each density distribution weighted by their density in the mixture model. The geometric mean was used for all receptor distributions (2,800 VEGFR1/Mixture, 1,250 VEGFR2/Mixture, 3,150 VEGFR1/D1, 950 VEGFR2/D1, 650 VEGFR1/D2, 1,500 VEGFR2/D2, 8,500 VEGFR1/D3, 2,300 VEGFR2/D3). The control reflects previously published VEGFR1 and VEGFR2 levels (1,100 VEGFR1/cell and 700 VEGFR2/cell) [23].
(TIF)

Table S1 Percent of each data set defined as outliers. Percent of each complete raw data set defined as outliers, where outliers are defined using low bin search or by removing all data 3 standard deviations (STD) above the mean. The largest difference in percent defined as outliers between low bin search and 3 STD is 7.59%.
(DOCX)

Author Contributions

Conceived and designed the experiments: JW PII. Performed the experiments: JW. Analyzed the data: JW PII. Contributed reagents/materials/analysis tools: JW PII. Wrote the paper: JW.

References

1. Saunders NA, Simpson F, Thompson EW, Hill MM, Endo-Munoz L, et al. (2011) Role of intratumoural heterogeneity in cancer drug resistance: Molecular and clinical perspectives. EMBO Mol Med 4: 675–684.
2. He B, Baird R, Butera R, Datta A, George S, et al. (2013) Grand challenges in interfacing engineering with life sciences and medicine. IEEE T Bio-Med Eng 60: 589–598.
3. Mac Gabhann F, Peirce SM (2010) Collateral capillary arterialization following arteriolar ligation in murine skeletal muslce. Microcirculation 17: 333–347.
4. Cruts M, Hendriks L, Van Broeckhoven C (1996) The presenilin genes: A new gene family involved in alzheimer disease pathology. Hum Mol Genet 5 Spec No: 1449–1455.
5. de Brabander H, van Hende J, Batjoens P, Hendriks L, Raus J, et al. (1994) Endogenic nortestosterone in cattle? Analyst 119: 2581–2585.
6. Hood JD (2002) Tumor regression by targeted gene delivery to the neovasculature. Science 296: 2404–2407.
7. Sugiyama Y, Kawabata I, Sobue K, Okabe S (2005) Determination of absolute protein numbers in single synapses by a GFP-based calibration technique. Nat Meth 2: 677–684.
8. Chiu CS, Jensen K, Sokolova I, Wang D, Li M, et al. (2002) Number, density, and surface/cytoplasmic distribution of GABA transporters at presynaptic structures of knock-in mice carrying GABA transporter subtype 1-green fluorescent protein fusions. J Neurosci 22: 10251–10266.
9. Hanley WD, Napier SL, Burdick MM, Schnaar RL, Sackstein R, et al. (2006) Variant isoforms of CD44 are P- and L-selectin ligands on colon carcinoma cells. FASEB J 20: 337–339.
10. Imoukhuede PI, Popel AS (2011) Quantification and cell-to-cell variation of vascular endothelial growth factor receptors. Experimental Cell Research 317: 955–965.
11. Imoukhuede PI, Popel AS (2012) Expression of VEGF receptors on endothelial cells in mouse skeletal muscle. PLoS One 7: e44791.

12. Imoukhuede PI, Dokun AO, Annex BH, Popel AS (2013) Endothelial cell-by-cell profiling reveals temporal dynamics of VEGFR1 and VEGFR2 membrane-localization following murine hindlimb ischemia. Am J Physiol Heart Circ Physiol 304: H1085–93.
13. Imoukhuede PI, Popel AS (2014) Quantitative fluorescent profiling of VEGFRs reveals tumor cell and endothelial cell heterogeneity in breast cancer xenografts. Cancer Medicine 3: 225–44.
14. Iyer SB, Bishop JE, Abrams B, Maino VC, Ward AJ, et al. (1997) QuantiBRITE: A new standard for PE fluorescence quantitation. BD Biosciences 23-3496-00.
15. Pannu KK, Joe ET, Iyer SB (2001) Performance evaluation of quantiBRITE phycoerythrin beads. Cytometry 45: 250–258.
16. Lee-Montiel FT, Imoukhuede PI (2013) Engineering quantum dot calibration standards for quantitative fluorescent profiling. J Mater Chem B 1: 6434–6441.
17. Boedigheimer MJ, Ferbas J (2008) Mixture modeling approach to flow cytometry data. Cytom Part A 73A: 421–429.
18. Le Meur N (2013) Computational methods for evaluation of cell-based data assessment - bioconductor. Curr Opin Biotech 24: 105–111.
19. Schoeberl B, Eichler-Jonsson C, Gilles ED, Muller G (2002) Computational modeling of the dynamics of the MAP kinase cascade activated by surface and internalized EGF receptors. Nature Biotech 20: 370–375.
20. Saterbak A, Kuo SC, Lauffenburger DA (1993) Heterogeneity and probabilistic binding contributions to receptor-mediated cell detachment kinetics. Biophys J 65: 243–252.
21. Stefanini MO, Wu FT, Mac Gabhann F, Popel AS (2008) A compartment model of VEGF distribution in blood, healthy and diseased tissues. BMC Syst Biol 2.
22. Stefanini MO, Wu FT, Mac Gabhann F, Popel AS (2010) Increase of plasma VEGF after intravenous administration of bevacizumab is predicted by a pharmacokinetic model. Cancer Res 70: 9886–9894.
23. Finley SD, Engel-Stefanini MO, Imoukhuede PI, Popel AS (2011) Pharmacokinetics and pharmacodynamics of VEGF-neutralizing antibodies. BMC Syst Biol 5: 193.
24. Williams CK, Li J, Murga M, Harris AL, Tosato G (2005) Up-regulation of the notch ligand delta-like 4 inhibits VEGF-induced endothelial cell function. Blood 107: 931–939.
25. Henderson AM, Wang SJ, Taylor AC, Aitkenhead M, Hughes CC (2001) The basic helix-loop-helix transcription factor HESR1 regulates endothelial cell tube formation. J Biol Chem 276: 6169–6176.
26. Leys C, Ley C, Klein O, Bernard P, Licata L (2013) Detecting outliers: Do not use standard deviation around the mean, use absolute deviation around the median. J Exp Soc Psychol 49: 764–766.
27. McLachlan G, Peel D (2004) Finite mixture models. Canada: John Wiley & Sons.
28. Zhu X, Zhang S, Zhao H, Cooper RS (2002) Association mapping, using a mixture model for complex traits. Genet Epidemiol 23: 181–196.
29. Zhang L, Gove JH, Liu C, Leak WB (2001) A finite mixture of two weibull distributions for modeling the diameter distributions of rotated-sigmoid, uneven-aged stands. Can J Forest Res 31: 1654–1659.
30. Schulz-Trieglaff O, Machtejevans E, Reinert K, Schluter H, Thiemann J, et al. (2009) Statistical quality assessment and outlier detection for liquid chromatography-mass spectrometry experiements. BioData Mining 2.
31. Eo SH, Pak D, Choi J, Cho HJ (2012) Outlier detection using projection quantile regression for mass spectrometry data with low replication. BMC Res Notes 5: 236.
32. Ghosh D, Chinnaiyan AM (2009) Genomic outlier profile analysis: Mixture models, null hypotheses, and nonparametric estimation. Biostatistics 10: 60–69.
33. Bashashati A, Brinkman RR (2009) A survey of flow cytometry data analysis methods. Adv Bioinformat 2009: Article ID 584603.
34. Lugli E, Roederer M, Cossarizza A (2010) Data analysis in flow cytometry: The future just started. Cytom Part A 77A: 705–713.
35. Aghaeepour N, Nikolic R, Hoos HH, Brinkman RR (2011) Rapid cell population identification in flow cytometry data. Cytom Part A 79A: 6–13.
36. Walther G, Zimmerman N, Moore W, Parks D, Meehan S, et al. (2009) Automatic clustering of flow cytometry data with density-based merging. Adv Bioinformat 2009: Article ID 686759.
37. Roederer M, Treister A, Moore W, Herzenberg LA (2001) Probability binning comparison: A metric for quantitating univariate distribution differences. Cytometry 45: 37–46.
38. Seely JF, Birkes D, Lee Y (1997) Characterizing sums of squares by their distributions. Am Stat 51: 55–58.
39. Kumanogoh A, Kikutani H (2013) Immunological functions of the neuropilins and plexins as receptors for semaphorins. Nat Rev Immunol 13: 802–814.
40. Serini G, Valdembri D, Zanivan S, Morterra G, Burkhardt C, et al. (2003) Class 3 semaphorins control vascular morphogenesis by inhibiting integrin function. Nature 424: 391–397.
41. Tan WH, Popel AS, Mac Gabhann F (2013) Computational model of Gab1/2-dependent VEGFR2 pathway to akt activation. PLoS One 8: e67438.
42. Kanno S, Oda N, Abe M, Terai Y, Ito M, et al. (2000) Roles of two VEGF receptors, flt-1 and KDR, in the signal transduction of VEGF effects in human vascular endothelial cells. Oncogene 19: 2138–2146.
43. Yu J, Bian D, Mahanivong C, Cheng RK, Zhou W, et al. (2004) p38 mitogen-activated protein kinase regulation of endothelial cell migration depends on urokinase plasminogen activator expression. J Biol Chem 279: 50446–50454.
44. Berthaut A, Mirshahi P, Benabbou N, Azzazene D, Bordu C, et al. (2009) Vascular endothelial growth factor receptor-1 (VEGFR-1) expression in human corneal fibroblast decreased with age. Mol Vis 15: 1997–2007.
45. Swano A, Iwai S, Sakurai Y, Ito M, Shitara K, et al. (2001) Flt-1, vascular endothelial growth factor receptor 1, is a novel cell surface marker for the lineage of monocyte-macrophages in humans. Blood 97: 785–791.
46. Shibuya M (2006) Vascular endothelial growth factor receptor-1 (VEGFR-1/flt-1): A dual regulator for angiogenesis. Angiogenesis 9: 225–230.
47. Clauss M, Gerlach M, Gerlach H, Brett J, Wang F, et al. (1990) Vascular permeability factor: A tumor-derived polypeptide that induces endothelial cell and monocyte procoagulant activity, and promotes monocyte migration. J Exp Med 172: 1535–1545.
48. Ferrara N, Gerber HP, LeCouter J (2003) The biology of VEGF and its receptors. Nat Med 9: 669–676.
49. Olsson AK, Dimberg A, Kreuger J, Claesson-Welsh L (2006) VEGF receptor signalling - in control of vascular function. Nat Rev Mol Cell Biol 7: 359–371.
50. Kearney JB, Kappas NC, Ellerstrom C, DiPaola FW, Bautch VL (2004) The VEGF receptor flt-1 (VEGFR-1) is a positive modulator of vascular sprout formation and branching morphogenesis. Blood 103: 4527–4535.
51. Quinn G, Ochiya T, Terada M, Yoshida T (2000) Mouse flt-1 promoter directs endothelial-specific expression in the embyroid body model of embryogenesis. Biochem Bioph Res Co 276: 1089–1099.
52. Sato T, Amano H, Ito Y, Eshima K, Minamino T, et al. (2014) Vascular endothelial growth factor receptor 1 signaling facilitates gastric ulcer healing and angiogenesis through the upregulation of epidermal growth factor expression on VEGFR1(+)CXCR4 (+) cells recruited from bone marrow. J Gastroenterol 49: 455–469.
53. Hiratsuka S, Nakamura K, Iwai S, Murakami M, Itoh T, et al. (2002) MMP9 induction by vascular endothelial growth factor receptor-1 is involved in lung specific metastasis. Cancer Cell 2: 289–300.
54. Jin K, Zhu Y, Sun Y, Mao XO, Xie L, et al. (2002) Vascular endothelial growth factor (VEGF) stimulates neurogenesis in vitro and in vivo. PNAS 99: 11946–11950.
55. Maurer MH, Tripps WKC, Feldmann RE Jr, Kuschinsky W (2003) Expression of vascular endothelial growth factor and its receptors in rat neural stem cells. Neurosci Lett 344: 165–168.
56. Mac Gabhann F, Popel AS (2007) Interactions of VEGF isoforms with VEGFR-1, VEGFR-2, and neuropilin in vivo: A computational model of human skeletal muscle. Am J Physiol Heart Circ Physiol 292: H459.
57. Lorch M, Booth PJ (2004) Insertion kinetics of a denatured α helical membrane protein into phospholipid bilayer vesicles. J Mol Biol 344: 1109–1121.
58. Nagy JA, Chang SH, Shih SC, Dvorak AM, Dvorak HF (2010) Heterogeneity of the tumor vasculature. Semin Thromb Hemost 36: 321–331.
59. Nagy JA, Dvorak HF (2012) Heterogeneity of the tumor vasculature: The need for new tumor blood vessel type-specific targets. Clin Exp Metastasis 29: 657–662.
60. De Bock K, Cauwenberghs S, Carmeliet P (2011) Vessel abnormalization: Another hallmark of cancer? molecular mechanisms and therapeutic implications. Curr Opin Genet Dev 21: 73–79.
61. Kushner EJ, Bautch VL (2013) Building blood vessels in development and disease. Curr Opin Hematol 20: 231–236.
62. Singh DK, Ku CJ, Wichaidit C, Steininger RJ 3rd, Wu LF, et al. (2010) Patterns of basal signaling heterogeneity can distinguish celllular populations with different drug sensitivities. Mol Syst Biol 6: 369.
63. Slack MD, Martinez ED, Wu LF, Altschuler SJ (2008) Characterizing heterogeneous cellular responses to perturbations. PNAS 105: 19306–19311.
64. Yarden Y SJ (1987) Self-phosphorylation of epidermal growth factor receptor: Evidence for a model of intermolecular allosteric activation. Biochem 26: 1434–1442.
65. Posner I, Engel M, Levitzki A (1992) Kinetic model of the epidermal growth factor (EGF) receptor tyrosine kinase and a possible mechanism of its activation by EGF. J Biol Chem 267: 20638–20647.
66. Resat H, Ewald JA, Dixon DA, Wiley HS (2003) An integrated model of epidermal growth factor receptor trafficking and signal transduction. Biophys J 85: 730–743.
67. Oda K, Matsuoka Y, Funahashi A, Kitano H (2005) A comprehensive pathway map of epidermal growth factor receptor signaling. Mol Syst Biol 1: 2005.0010.
68. Tan WH, Popel AS, Mac Gabhann F (2013) Computational model of VEGFR2 pathway to ERK activation and modulation through receptor trafficking. Cell Signal 25: 2496–2510.
69. Mac Gabhann F, Popel AS (2004) Model of competitive binding of vascular endothelial growth factor and placental growth factor to VEGF receptors on endothelial cells. Am J Physiol Heart Circ Physiol 286: H153–H164.
70. Mac Gabhann F, Popel AS (2007) Dimerization of VEGF receptors and implications for signal transduction: A computational study. Biophys Chem 128: 125–139.
71. Finley SD, Popel AS (2013) Effect of tumor microenvironment on tumor VEGF during anti-VEGF treatment: Systems biology predictions. J Nat Cancer Institute 105: 802–811.
72. Finley SD, Dhar M, Popel AS (2013) Compartment model predicts VEGF secretion and investigates the effects of VEGF Trap in tumor-bearing mice. Front Oncol 3: 196.

73. Finley SD, Popel AS (2012) Predicting the effects of anti-angiogenic agents targeting specific VEGF isoforms. AAPS J 14: 500–509.
74. Hicklin DJ, Ellis LM (2005) Role of the vascular endothelial growth factor pathway in tumor growth and angiogenesis. J Clin Oncol 23: 1011–1027.
75. Ellis LM, Hicklin DJ (2008) VEGF-targeted therapy: Mechanisms of anti-tumour activity. Nat Rev Cancer 8: 579–591.
76. Kut C, Mac Gabhann F, Popel AS (2007) Where is VEGF in the body? A meta-analysis of VEGF distribution in cancer. Br J Cancer 97: 978–985.
